# Optimization of an Injectable, Resorbable, Bioactive Cement Able to Release the Anti-Osteoclastogenic Biomolecule ICOS-Fc for the Treatment of Osteoporotic Vertebral Compression Fractures

**DOI:** 10.3390/biom13010094

**Published:** 2023-01-02

**Authors:** Federica Banche-Niclot, Ilaria Corvaglia, Caterina Cavalera, Elena Boggio, Casimiro Luca Gigliotti, Umberto Dianzani, Antzela Tzagiollari, Nicholas Dunne, Antonio Manca, Sonia Fiorilli, Chiara Vitale-Brovarone

**Affiliations:** 1 Department of Applied Science and Technology, Politecnico di Torino, Corso Duca Degli Abruzzi 24, 10129 Torino, Italy; 2 NOVAICOS s.r.l.s., Via Amico Canobio 4/6, 28100 Novara, Italy; 3 Department of Health Sciences, Università del Piemonte Orientale, Via Solaroli 17, 28100 Novara, Italy; 4 Centre for Medical Engineering Research, School of Mechanical and Manufacturing Engineering, Dublin City University, D09 NA55 Dublin, Ireland; 5 Biodesign Europe, Dublin City University, D09 NA55 Dublin, Ireland; 6 Department of Radiology, Candiolo Cancer Institute, FPO-IRCCS, 10060 Torino, Italy; 7 National Interuniversity Consortium of Materials Science and Technology, RU Politecnico di Torino, 50121 Firenze, Italy

**Keywords:** injectable cement, calcium sulphate, mesoporous bioactive glasses, PLGA nanoparticles, ICOS-Fc, bone, osteoporosis, tissue regeneration

## Abstract

Vertebral compression fractures are typical of osteoporosis and their treatment can require the injection of a cement through a minimally invasive procedure to restore vertebral body height. This study reports the development of an injectable calcium sulphate-based composite cement able to stimulate bone regeneration while inhibiting osteoclast bone resorption. To this aim, different types of strontium-containing mesoporous glass particles (Sr-MBG) were added to calcium sulphate powder to impart a pro-osteogenic effect, and the influence of their size and textural features on the cement properties was investigated. Anti-osteoclastogenic properties were conferred by incorporating into poly(lactic-co-glycolic)acid (PLGA) nanoparticles, a recombinant protein able to inhibit osteoclast activity (i.e., ICOS-Fc). Radiopaque zirconia nanoparticles (ZrO_2_) were also added to the formulation to visualize the cement injection under fluoroscopy. The measured cement setting times were suitable for the clinical practice, and static mechanical testing determined a compressive strength of ca. 8 MPa, comparable to that of human vertebral bodies. In vitro release experiments indicated a sustained release of ICOS-Fc and Sr^2+^ ions up to 28 days. Overall, the developed cement is promising for the treatment of vertebral compression fractures and has the potential to stimulate bone regeneration while releasing a biomolecule able to limit bone resorption.

## 1. Introduction

Approximately 200 million people worldwide are suffering from osteoporosis (OP) [1], a metabolic bone disease caused by an excessive osteoclast (OC) resorption activity that increases the risk of fracture. Particularly, vertebral compression fractures are one of the most frequent [2] and severely affect the life quality of patients causing intensive pain, kyphotic deformation of the column, immobility, swallowing and breathing difficulties and they considerably increase the rate of mortality [3]. The choice of the most appropriate treatment for these fractures depends on the nature of the fracture itself and the state of the health of the patient. Conservative treatments foresee the administration of drugs and rest periods followed by the application of orthoses and hyperextension braces; however, they are largely ineffective. Traditional surgical procedures involve the decompression and stabilization of the fractures through the implantation of screws and fixing plates. Nevertheless, this approach inevitably exposes the patients to risks and revision operations, as well as, in most cases, the low quality of osteoporotic bone that prevents proper healing. To allay these limitations, minimally invasive surgical techniques, such as vertebroplasty (VP), have been increasingly exploited since the early 1980s due to their higher safety, lower invasiveness and costs compared to standard surgery and especially due to the speed of intervention (usually takes about 1 h to complete), allowing fast relief of pain and mobility restoration.

VP involves the percutaneous injection under fluoroscopic guidance of a viscous cement through an 11–13 Gauge needle into the weakened vertebral body [4,5]. The biomaterials used during this surgical procedure consist of a dry powder phase and a liquid phase, which, when mixed together, result in a viscous paste that can be directly injected into the fractured site, where it will harden. The hardening of such cements within the body allows the stabilization of the fractured vertebra, preventing micromotion and thus reducing the extent of the patient’s pain [6].

A variety of cements have been developed for spinal stabilization, which should possess specific characteristics to be used in the clinic, as well as provide successful performance: i.e., (i) Biocompatibility; (ii) Injectability and cohesion of the extruded paste to prevent leaks; (iii) Self-setting properties enabling an adequate timeframe by which it can be injected; (iv) Suitable mechanical properties ideally matching those of the host tissue (compressive strength of bone ranging between 2–12 MPa [7]) with the aim of reducing the possibility of structural collapses; (v) Sufficient radiopacity to enable the correct distribution of the material within the vertebrae and verify the absence of any cement leakages, achieved by the introduction of zirconia (ZrO_2_) or barium sulphate (BaSO_4_) particles or the use of an iodine-based element. In addition, bioactive and resorbable features are recommended, offering certain benefits for full tissue integration, and reducing the invasiveness of the implant.

Three types of injectable cements for VP are currently on the market that meet most of these requirements: acrylic cement as inert one, calcium phosphates and calcium sulphates cements as resorbable materials, and composite cements. To date, the most commonly used injectable cement is the acrylic one based on poly(methyl methacrylate) (PMMA). Although PMMA cements exhibit good mechanical properties, they present several intrinsic drawbacks, including lack of osteointegration, leaching of toxic unreacted monomers into the bloodstream, fast polymerization kinetics that lead to a short handling time and temperatures up to 110 °C that can be reached during the setting phase due to their exothermal polymerization reaction, which can cause the necrosis of the surrounding tissue [8]. These disadvantages have directed the interest of many researchers towards alternative biomaterials.

As a promising option, calcium sulphate [9] or calcium phosphates [10,11] bioresorbable cements (CSCs and CPCs, respectively) have been developed. These biomaterials actively interact with the body due to their excellent biocompatibility and their similar chemical composition to the inorganic component of human bone tissue [12]. Moreover, these resorbable biomaterials spontaneously harden at a physiological temperature of 37 °C following a dissolution-precipitation reaction of calcium salts. In particular, once mixed with water, CSH powder transforms into its hydrated form known as calcium sulphate dihydrate (CSD) and results in a paste-like material that can be directly injected through a VP needle (13–15 Gauge) into the fractured vertebral body. The obtained paste-like materials harden due to the precipitation of CSD from the aqueous solution, following the reaction reported below:

(1) $${\mathrm{CaSO}}_{4}\cdot \frac{1}{2}H_{2}O\;\overset{+1.5{\;H}_{2}O}{\to }{\mathrm{CaSO}}_{4}\cdot 2H_{2}O$$

This precipitation occurs as CSD is less soluble in water than CSH: 0.20 *w*/*w*% CSD dissolves at room temperature when mixed with water compared to 0.65 *w*/*w*% for CSH [13,14].

The main advantages of resorbable cements over their acrylic counterpart are related to their degradation, ruled by the bioerosion process, and their bone integration. Furthermore, due to their ability to release a high amount of calcium ions during degradation, they exhibit a bioactive behavior that confers both osteoconductive and osteoproductive properties, which can better support bone tissue regeneration. In fact, in vivo experiments demonstrate spontaneous precipitation of a layer of hydroxyapatite (HA) at the implantation site [15,16]. CSCs can represent a preferable solution to CPCs since they have long been used in vivo and because CPCs showed a degradation rate slower than the new bone formation, which limits their clinical use [17,18]. One such material is Cerament^®^ (BONESUPPORT, Lund, Sweden), a patented medical-grade calcium sulphate bone-graft substitute and bone void filler consisting of 60% wt of calcium sulphate hemihydrate particles enriched with 40% wt of nanoHA particles and an iodine-based liquid phase to confer radiopacity. In addition, both CSCs and CPCs are more brittle under mechanical loading when compared to PMMA cement exhibiting a lower compressive strength value. In this regard, the design of multiphasic composite materials (e.g., Cerament^®^) represents a valid field of research to overcome the limitations due to the matrix properties.

Generally, resorbable composite-based cements are based on a calcium phosphate or sulphate as the matrix material and the incorporation of inorganic or organic dispersed phases to modulate the degradation kinetics, enhance the bioactivity and improve the mechanical properties. A very promising strategy to improve the bioactivity features is the introduction of mesoporous bioactive glasses (MBG) within the ceramic matrix. This class of biomaterials gained increasing attention over the years due to their excellent biocompatibility and high reactivity, as well as the possibility to incorporate therapeutic ions in their composition alongside their ability to quickly create stable bonds with the bone tissue thanks to their spontaneous ability to form HA at the interface with the physiological environment [19]. Moreover, their mesopores allow for the hosting of relatively large levels of biomolecules and drugs reaching an adsorption efficiency greater than 80% [20,21] and are amenable to surface functionalization. Among the several ions that can be included in the MBG compositions, strontium is frequently used to confer pro-osteogenic ability to the material and it is currently exploited for the treatment of OP [22,23] due to its therapeutic effect on the metabolism of bone tissue. Indeed, it was broadly demonstrated that strontium can simultaneously regulate the activity of osteoblasts (OBs) and OCs by interacting with receptors sensitive to the presence of calcium (CaSR) and participating in the receptor activator of nuclear factor κ B (RANK)/RANK ligand (RANKL)/osteoprotegerin (OPG) signaling pathway [24,25,26]. Some research studies have suggested that Sr^2+^ ions can increase the secretion of OPG and inhibit that of RANKL by OBs interacting with the CaSR receptors on their cell membranes, resulting in a stimulation of OBs activity [27]. Conversely, the binding of CaSR receptors on OCs can induce their apoptosis [28].

Based on these considerations, the present research work reports the optimization of a resorbable radiopaque injectable composite cement for the stabilization of vertebral compression fractures made of calcium sulphate used as matrix and combined with ZrO_2_ nanoparticles and strontium-containing MBGs (Sr-MBG) to impart both radiopacity and pro-osteogenic features in an attempt to stimulate an appropriate bone remodeling response, favoring an effective healing. Different formulations have been tested varying the ratio between the resorbable matrix CSH and the inorganic phase Sr-MBG and comparing to controls: only CSH (100 CSH) and the CSH-based commercial reference Cerament^®^. In addition, the effects of two different types of Sr-MBG particles (i.e., one synthesized via a base-catalyzed sol-gel method (Sr-MBG-SG) and the other obtained through an aerosol-assisted spray-drying technology (Sr-MBG-SD)) on the properties of the final cement were investigated with the intention of developing the optimal cement formulation in terms of injectability, setting times, mechanical properties and resorption rate.

Finally, considering the purpose of treating osteoporotic fractures, an anti-osteoclastogenic stimulus has been introduced in the optimized cement formulation. To this aim, the recombinant protein ICOS-Fc has been employed due to its demonstrable ability to slow down OC activity both in vitro and in vivo [29,30]. ICOS-Fc is a chimeric protein exhibiting two peptide sequences in its fragment antigen-binding (F_ab_) region, which are the extracellular portion of the transmembrane protein ICOS. The latter is mainly expressed on T cells, it is able to recognize the ICOS ligand (ICOSL) and presents on the surface of several antigen-presenting cells such as B cells, dendritic cells and many tumor cells [31,32,33,34]. The ICOS–ICOSL interaction is well-known and widely studied in immunology; however, a recent study reported that this interaction is also involved in the bone remodeling process since OCs (which derive from the monocyte lineage, similar to dendritic cells) display ICOSL on their membrane. In particular, Gigliotti et al. demonstrated that the triggering of ICOSL expressed in OCs by the recombinant soluble form of ICOS (ICOS-Fc) inhibits the differentiation of monocytic cells towards the osteoclastic phenotype and the activity of mature OCs [30].

Despite these remarkable characteristics, it is known that the free dispersion of biomolecules within biomaterials is commonly associated with rapid clearance and insufficiently localized release [35,36]. Hence, the administration of high doses to reach effective concentrations is frequently required, resulting in the increase in the toxicity risk. Therefore, there is a high demand for delivery vehicles that can provide adequate, sustained, and localized presentation of biomolecules in a time-dependent manner. In this context, there have been many attempts in the past to achieve controlled release kinetics using engineered delivery systems able to mimic, recapitulate and manipulate the signaling processes of tissue regeneration on a similar spatio-temporal scale that could facilitate and enhance the regenerative process [37]. Among the most recent studies reported in the literature, more than 50% of the research efforts relate to the development of polymeric nanoparticles used as small reservoirs, where the biomolecule is embedded into a polymeric matrix serving as diffusional barrier [38]. They present ultra-small dimensions (1–500 nm in size) with a large surface-area-to-mass ratio and high reactivity and offer a functionalized structure [39,40]. A variety of polymers have been exploited for the manufacturing of polymeric nanoparticles; among them the most frequently used is poly(lactic-co-glycolic) acid (PLGA) thanks to the proven in vivo biocompatibility and minimal systemic toxicity [41]. The most interesting feature of PLGA is its tunable degradation rate due to its chemical properties such as the initial molecular weight of the polymers, lactic-to-glycolic ratio (L:G), end-groups, crystallinity, stereochemistry, as well as the exposure time to liquid and the storage temperature [41]. In this scenario, in the present study the authors synthetized PLGA particles with nanometric dimensions able to encapsulate and release functional ICOS-Fc (ICOS_PLGA). Afterwards, ICOS_PLGA was incorporated into the optimal cement formulation to provide an anti-osteoclastogenic stimulus. The obtained injectable cement was fully characterized and the ability of polymeric nanoparticles to modulate the ICOS-Fc release kinetics compared to its direct dispersion into the cement paste has been investigated.

To the best of the authors’ knowledge, the proposed cement is the first one that adds pro-osteogenic and anti-osteoclastogenic properties into an injectable resorbable formulation. Cements currently on the market (e.g., Cerament^®^) are designed for the stabilization of weakened vertebrae and can provide anti-inflammatory cues by the incorporation of drugs [42,43]. The authors believe that the developed formulation is attractive since it goes significantly beyond the current clinical approaches through the synergistic combination of bone regeneration stimulation with the reduction in bone loss due to the action of therapeutic ions and the release of ICOS-Fc.

## 2. Materials and Methods

### 2.1. Preparation of the Injectable Cement

The cement was obtained by mixing a dry powder phase with a liquid phase creating a paste-like material that can be directly injected into the vertebral fracture site through a 13 Gauge needle, the most commonly used in percutaneous VP usually inserted via transpedicular approach. Double-distilled water (ddH_2_O) was used as liquid phase. The mixed powder phase of the cement consisted of the combination of particles of α-type calcium sulphate hemihydrate (α-CSH; GC FUJIROCK EP Classic, GC EUROPE, San Giuliano Milanese, Italy), used as resorbable matrix, with commercially available ZrO_2_ nanoparticles (ANHUI RENCHENG TECHNOLOGY CO, Hefei, China) to confer the suitable grade of radiopacity. Furthermore, two different types of Sr-MBG particles were separately included within the formulation to impart bioactive and pro-osteogenic features. These particles, containing 10% mol of strontium (Si/Ca/Sr = 85/5/10), were produced following protocols previously reported by the authors [44]: a base-catalyzed sol-gel synthesis (Sr-MBG_SG) resulting in nanometric particles with high textural properties (i.e., specific surface area; SSA) and an aerosol-assisted spray-drying method (Sr-MBG-SD) leading to micrometric particles with spherical morphology. The structural and morphological characteristics of commercially available ZrO_2_ nanoparticles, as well as of synthesized Sr-MBG are reported in Figure S1.

The formulation of the cement was finely tuned adjusting the ratio between the resorbable matrix CSH and Sr-MBG particles in order to achieve the best performance in terms of injectability, setting times, mechanical properties and in vitro behavior. The tested formulations are listed in Table 1. The amount of the radiopaque component was maintained at 5% vol. for all the developed formulations. The injectable paste was obtained by manually mixing the powder phase with an appropriate amount of ddH_2_O for 90 s, setting at a room temperature (RT) of 20 °C, which is the typical temperature within an operating room (OR). The characteristics of the developed formulations were compared with two controls: CSH alone, used to highlight the effects of Sr-MBG on the developed cement, and Cerament^®^ (BONESUPPORT, Lund, Sweden) as reference commercial material that was prepared according to the manufacturer’ instructions.

### 2.2. Preparation of the Anti-Osteoclastogenic Injectable Cement

Considering the final purpose of developing a biomaterial for the treatment of osteoporotic fractures, an aqueous solution containing 5% wt of ICOS-Fc (NOVAICOS s.r.l.s., Novara, Italy) was used as liquid phase. Alternatively, nanoparticles of PLGA containing ICOS-Fc (ICOS_PLGA) were incorporated in the optimized cement formulation to provide a more sustained release of the anti-osteoclastic cue.

#### Synthesis of ICOS-Fc-Containing PLGA Nanoparticles

ICOS_PLGA were produced through a double-emulsion method adapting a procedure previously reported by the authors [45].

Briefly, an aqueous ICOS-Fc (human, NOVAICOS s.r.l.s., Novara, Italy) solution with a concentration of 20 ng/μL was added dropwise into an organic solution of PLGA (50:50, Mw 25,000; Sigma-Aldrich, Milan, Italy) at a concentration of 60 mg/mL, and the mixture was then sonicated (Sonoplus GM3200, Bandelin, Berlin, Germany) in ice for 1 min to yield a water-in-oil emulsion and allow ICOS-Fc incorporation. Next, 5 mL of 1% wt poly(vinyl alcohol) (PVA, Mw 30,000–70,000 Da, 87–90% hydrolyzed; Sigma-Aldrich, Milan, Italy) aqueous solution was added to the primary water-in-oil emulsion and further emulsified for 2 min to stabilize the nanoparticles’ formation. The organic solvent was then allowed to evaporate overnight at RT, and finally, the ICOS_PLGA nanoparticles were washed several times with sterile ddH_2_O, resuspended in sterile ddH_2_O and stored at −20 °C.

The synthesized nanoparticles were characterized in terms of morphology, size distribution and physicochemical features. For morphological analysis, the field-emission scanning electron microscope instrument (FE-SEM; ZEISS MERLIN instrument, Oberkochen, Germany) working at 1.5 kV and 3.0 kV acceleration voltage was used. For this investigation, about 10 mg of ICOS_PLGA were dispersed in 5 mL of ddH_2_O using an ultrasonic bath (Digitec DT 103H, Bandelin) for 10 min and the resulting suspension was dropped on a copper grid (3.05 mm in diameter, 200 mesh, TAAB, Aldermaston, UK), allowed to dry and successively placed onto a conductive carbon tape adhered on an aluminum-stub. A 7 nm thick platinum sputtered coating was used to increase sample conductivity. The size distribution of the nanoparticles was assessed using dynamic light scattering (DLS; Zetasizer Nano ZS90 instrument, Malvern Instruments Inc., Malvern, UK) by analyzing 1 mL of a water suspension of ICOS_PLGA with a concentration of 0.1 mg/mL. The test was performed in triplicate (15 runs for 3 measurements) at 25 °C by setting the viscosity and refraction index to the reference values of water. Furthermore, attenuated total reflectance infrared spectroscopy (ATR-FTIR) was used to assess the physicochemical features of the ICOS_PLGA nanoparticles by using the Equinox 55 spectrometer (Bruker, Ettlingen, Germany) equipped with an MCT cryodetector and an ATR accessory. Samples were frozen at −20 °C and lyophilized for 24 h before analysis. Spectra were collected over a range of wavenumbers from 4000 and 600 cm^−1^ at a 4 cm^−1^ resolution using 32 scans. The spectra were reported after background subtraction, baseline correction, and smoothing (11 points) using OPUS software (Bruker, Ettlingen, Germany).

**Figure 1:**
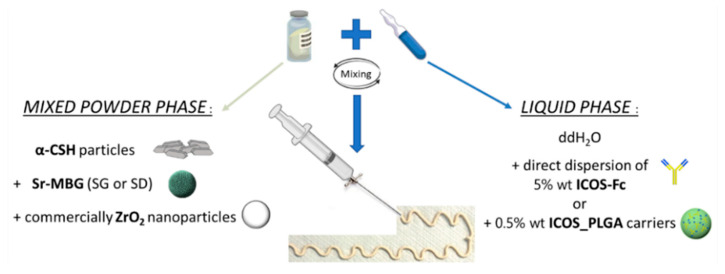
Schematic representation of the developed anti-osteoclastogenic formulations.

Then, based on the therapeutic concentration of directly administered ICOS-Fc reported in the literature [30], as well as the performances of the synthetized ICOS-Fc carrier, 0.5% wt of ICOS_PLGA was added to the cement (Figure 1). The polymeric nanoparticles were dispersed in the liquid phase in order to guarantee their stability and to achieve a better dispersion of them throughout the CSH matrix during mixing. The anti-osteoclastogenic injectable paste was obtained as presented above and as schematically represented in Figure 1.

### 2.3. Injectability Optimization and Morphological Assessement

Firstly, the liquid-to-powder (L/P) ratio of each tested formulation was optimized to enable the injection of at least 75% vol. of the paste through a 13 Gauge needle. The experiment was conducted by setting an RT of 20 °C to simulate the OR environmental mixing conditions. For the 100 CSH formulation and Cerament^®^, the L/P reported by the manufacturer was used (i.e., 0.20 and 0.40, respectively). Moreover, the consistency of the extruded filament after hardening was qualitatively examined by applying a uniaxial compressive load to the dry extruded filament after a setting step of 24 h at 37 °C.

The surface morphology of all investigated biomaterials and the dispersion of Sr-MBG and ZrO_2_ particles within the CSD matrix were analyzed by means of FE-SEM at 3 kV and backscattering mode. Before analysis, solid samples were prepared by filling a cylindrical mold (dimensions of 13.5 mm in diameter (D) and 5 mm in height (H)) with the cement paste, let to harden for 24 h at 37 °C, 100% humidity. Afterwards, the set samples were fixed onto a conductive carbon tape adhered to an aluminum-stub and, prior to the investigation, coated with a 7 nm thick layer of platinum.

### 2.4. Radiopacity Assessement

The adequate grade of radiopacity and its distribution along the cement were analyzed through fluoroscopy imaging and micro-computed tomography (Micro-CT) investigation, respectively. In detail, fluoroscopy was performed to confirm that the percentage of the radiopaque element included in the formulations was sufficient for clinical applications compared to those of commercial reference. A clinical instrument was employed and cylindrical specimens of 5 × 3 mm (D × H) were created as previously described. The distribution of the radiopaque element throughout the volume of developed cements and Cerament^®^ was evaluated by means of a Micro-CT instrument (SkyScan 1272; Bruker, Ettlingen, Germany). About 1.5 mL of paste was used to create 13.5 × 10 mm solid samples, subsequently scanned with a resolution of 7 μm and applying a voltage of 100 kV, using a copper filter having a thickness of 0.11 mm to eliminate the less energetic fraction of the X-ray beam. The samples were analyzed over 180°, selecting a rotation step of 0.3°. After the reduction in the artifacts resulting from the scanning process, the acquired images were reconstructed through the N-Recon software (Bruker, Ettlingen, Germany). Finally, through the 3D rendering software CTVox (Bruker, Ettlingen, Germany), a 3D model of the sample volume was obtained and the specimens were stained according to the density of the different components by means of an RGB color code to evaluate the distribution of the radiopaque element (e.g., ZrO_2_ nanoparticles for the developed cement and iohexol for the Cerament^®^).

### 2.5. Setting Times Evaluation

The initial and final setting times of the cement (i.e., the timeframes during which the cement can be injected and after which it is fully hardened, respectively) were determined in accordance with the ASTM-C266-04 standard and by using the Gillmore apparatus. The measurements were conducted using cylindrical samples (13.5 × 10 mm, D × H) every 2 min starting from the end of the mixing and were repeated for 3 different samples, reporting the results as mean value ± standard deviation. The assessment of the initial setting time was performed at 20 °C to simulate the clinical conditions of operation, while the evaluation of the final setting time was completed at 37 °C, 100% humidity, with the aim of mimicking the physiological conditions of the site where the cement will be injected.

### 2.6. Mechanical Testing

Uniaxial mechanical testing was conducted in accordance with standard ISO 5833-2002 on cylindrical specimen (6 × 12 mm, D × H) using the Zwick Z5 Testing Machine (Zwick Roell, Leominster, UK) fitted with a 5 kN load cell. In particular, the samples were tested at two different time points, after 24 h (under wet conditions) and seven days (under dry conditions) from the end of mixing to assess the initial and post-hydrated mechanical behavior of the cement. Both types were placed in an incubator at 37 ± 1 °C under 100% (wet) and 0% (dry) humidity conditions. A compressive load was applied at a rate of displacement of 1 mm/min using a preload of 5 N at a room temperature of 23 ± 1 °C. Prior to the mechanical testing, the diameter of each specimen was measured. Each specimen was tested to failure to determine the compressive strength (i.e., maximum failure load/cross-sectional area of specimen). The compressive strength of each formulation was determined in triplicate and the data were reported as the mean value ± standard deviation.

### 2.7. Porosity Analysis

Moreover, the Micro-CT instrument was used to analyze the distribution of the porosity throughout the entire volume of the cement in order to correlate the influence of pores to the mechanical behavior. As described above, samples of 13.5 × 10 mm (D × H) were scanned with a resolution of 7 μm, applying a voltage of 100 kV and utilizing a copper filter with a thickness of 0.11 mm. After image reconstruction through the N-Recon software, the CTAn software (Bruker, Ettlingen, Germany) was utilized to evaluate the percentage of porosity of each sample and pore dimension. A custom-made analysis was performed by setting a threshold of 40, and morphological operations were made in order to remove speckles and noise from the images before executing the 3D analysis to evaluate the total porosity of the sample. Starting from the same custom-made morphological operations, the size distribution of pores was also evaluated by performing individual 3D analyses.

### 2.8. In Vitro Degradation Test

In vitro degradation tests were conducted in accordance with standard ISO 10993-14 by soaking cylindrical samples (13.5 × 10 mm, D × H) in a Tris-HCl solution (0.1 M, pH 7.4) at a concentration of 1 g of materials/20 mL of buffer. In detail, after a setting period of 24 h in an incubator at 37 °C (100% humidity), samples were weighed and measured in terms of height and diameter. Once immersed in the respective volume of Tris-HCl solution, the samples were placed in an orbital shaker (Excella E24, Eppendorf, Hamburg, Germany) at 37 °C with an agitation rate of 120 rpm until their complete degradation. Every 7 days, the Tris-HCl solution was fully refreshed and the samples corresponding to the specific time point were washed twice with ddH_2_O, dried in an oven at 70 °C for 2 h and their weight and dimension were recorded. The residual weight percentage (residual weight) of each sample was calculated as follows:

(2) $$\mathrm{Residual}\;\mathrm{weight}\;\left(\%\right)=\left(1-\frac{w_{o}-w_{t}}{w_{o}}\right)\times 100$$

where *w*_o_ is the initial weight of the sample (g) and *w*_t_ is the weight recorded in the specific time point t (g). The experiments were conducted in duplicate, and results were reported as mean ± standard deviation.

### 2.9. In Vitro Bioactivity Assessment

In vitro bioactivity tests were conducted to evaluate the apatite-forming ability of the developed cements achieved by the introduction of Sr-MBG within the formulations. To this aim, cylindrical samples (13.5 × 10 mm, D × H) were immersed in 100 mL of simulated body fluid (SBF), prepared following the protocol described in the literature [46], at 37 °C, 120 rpm up to 28 days in an orbital shaker (Excella E24, Eppendorf). The experiments were conducted in duplicate and the SBF solution was completely replaced every 7 days with a fresh solution to maintain the ion exchange active. At each time point (3 h, 1, 3, 7, 14, 21 and 28 days), the supernatant was withdrawn, the samples were washed three times with ddH_2_O and dried in an oven at 70 °C for 2 h. FE-SEM and X-ray Diffraction (XRD, X’Pert PRO diffractometer; Malvern Panalytical, Grovewood Road, United Kingdom) analyses were carried out on the collected samples to evaluate the apatite layer formation. In brief, samples for morphological investigation were prepared as described in Section 2.3, whereas XRD analysis was carried out in the 2ϴ degrees range of 15 and 70° by setting a current of 40 mA and a voltage of 40 kV.

### 2.10. Sr^2+^ Release in Vitro Evaluation

The concentration of Sr^2+^ ions released from the composite cement was evaluated under physiologic conditions (37 °C, pH 7.4) for up to 28 days by soaking 13.5 × 10 mm (D × H) solid samples in Tris-HCl solution at a concentration of 1 g of materials/20 mL of buffer. Briefly, samples were soaked at 37 °C with an agitation speed of 120 rpm (Excella E24, Eppendorf, Hamburg, Germany). At specific timepoints (3 h, 1, 3, 7, 14, 21 and 28 days), the supernatant was collected, stored at 4 °C until analysis and a complete refresh of the solution was performed. The Sr^2+^ concentration was measured through the inductively coupled plasma atomic emission spectrometry (ICP-AES) technique (iCPA RQ ICP-MS, Thermoscientific, Waltham, MA, USA) after appropriate aqueous dilutions of the collected supernatants (1:100) to not saturate the instrument detector. All the experiments were conducted in triplicate and results were reported as mean value ± standard deviation. Prior to release experiments, the concentration of strontium ions initially present in the CSH/Sr-MBG/ZrO_2_ cements was determined. For this purpose, 15 mg of cement were dissolved in a mixture of nitric and hydrofluoric acids, heated for 12 h at 70 °C and the resulting solutions were analyzed via ICP-AES analysis. The experiments were completed in triplicate and the results were reported as mean value ± standard deviation.

### 2.11. ICOS-Fc Release Kinetics Evaluation

The ability of the developed cement to release the anti-osteoclastogenic biomolecule, ICOS-Fc, was evaluated for up to 28 days under physiological conditions. Before this test, the amount of ICOS-Fc encapsulated into ICOS_PGLA nanoparticles and their ability to deliver the anti-osteoclastogenic biomolecule had been assessed.

Encapsulation efficiency tests on developed carriers were performed after the dissolution of the polymeric matrix to extract ICOS-Fc molecules. To this aim, the following extraction protocol was followed [47]. Briefly, 100 ng of dry ICOS_PLGA particles lyophilized (Lyovapor L-200 freeze-dryer, Büchi, Cornaredo, Italy) for 24 h were dissolved in 1 mL of dimethylsulfoxide (DMSO, >99%, Sigma-Aldrich, Milan, Italy) and the solution was stirred for 1 h at RT. Subsequently, 3 mL of 0.01 M HCl solution was added, and the mixture was stirred for a further hour. After centrifuging (Hermle Labortechnik Z326, Wehingen, Germany) at 10,000 rpm for 10 min at 10 °C to allow polymer precipitation, the supernatant was collected, flash-frozen with liquid nitrogen and lyophilized (Lyovapor L-200 freeze-dryer, Büchi, Cornaredo, Italy) over 24 h to concentrate ICOS-Fc, and then resuspended in ddH_2_O at a concentration suitable for the detection tests. The ICOS-Fc content was quantified using Micro BCA Protein Assay (Thermoscientific, Waltham, MA, USA) according to the manufacturer’s instructions. The percentage of encapsulation efficiency (EE) was expressed as the ratio of the actual value of total protein content to the theoretical value:

(3) $$\mathrm{EE}\;\left(\%\right)=\frac{\mathrm{amount}\;\mathrm{of}\;\mathrm{incorporated}\;\mathrm{ICOS}-\mathrm{Fc}}{\mathrm{initial}\;\mathrm{amount}\;\mathrm{of}\;\mathrm{ICOS}-\mathrm{Fc}}\times 100$$

The measurements were performed in triplicate and the EE% was reported as mean value ± standard deviation. Micro BCA Protein Assay on empty samples was used as negative control and subtracted.

The ability of ICOS_PLGA nanocarriers to release ICOS-Fc was studied under physiological conditions over 28 days and estimated by using Micro BCA Protein Assay. Briefly, ICOS_PLGA were dispersed in PBS, pH 7.4 with a concentration of 10 mg/mL and placed in an orbital shaker (Excella E24, Eppendorf, Hamburg, Germany) at 37 °C and 70 rpm. At indicated timepoints (1, 3, 7, 14, 21 and 28 days), samples were centrifuged (Hermle Labortechnik Z326, Hermle LaborTechnik GmbH, Wehingen, Germany) at 8500 rpm, 10 °C for 5 min. The entire volume was withdrawn and replaced with an equal amount of fresh release media. The collected samples were then stored at −20 °C until analysis. The measurements were performed in triplicate, and the cumulative percentages of released ICOS-Fc were reported as mean value ± standard deviation.

The cement with 5% wt of ICOS-Fc or 0.5% wt of ICOS_PLGA were produced as cylindrical specimens (13.5 × 5 mm, D × H), immersed in 20 mL PBS (37 °C, pH 7.4) and placed in an orbital shaker (Excella E24, Eppendorf, Hamburg, Germany) at 120 rpm. At specific timepoints (3 h, 1, 3, 7, 14, 21 and 28 days), the supernatant was collected, stored at −20 °C until analysis using Micro BCA Protein Assay and completely refreshed. The measurements were performed in triplicate and results were reported as mean value ± standard deviation.

## 3. Results

### 3.1. Injectability, Morphology and Radiopacity of the Composite Cement Formulations

The selection of the optimal L/P is a fundamental step in the development of an injectable cement since directly affects many properties of the material such as injectability, setting times as well as the mechanical strength [48,49]. Generally, all the optimized L/P allowed a percentage of injectability greater than 75% vol., which was considered suitable for the clinical application.

Overall, from the results reported in Table 2, it can be stated that decreasing the CSH content and concurrently increasing the % vol. of Sr-MBG led to a higher L/P in order to obtain an injectable paste. Higher L/P were recorded when Sr-MBG-SD were used due to their micrometric dimension and lower water uptake compared to Sr-MBG-SG due to lower exposed surface area (SSA of 85 m^2^/g and 465 m^2^/g, respectively [44]), which affects the compactness of the powder phase and the uptake of water during mixing, respectively.

Although all the tested formulations showed a high percentage of injectability, those with an Sr-MBG-SD content higher than 5% vol. led to a complete crumbling of the samples once hardened, which would result in the failure of the cement once subjected to a compressive load. Conversely, all the formulations containing Sr-MBG-SG and the 90CSH/5Sr-MBG-SD/5ZrO_2_ one did not show this negative aspect and were further investigated.

As far as the anti-osteoclastogenic formulation containing the polymeric nanoparticles is concerned, the morphology and the structural features of ICOS_PLGA were assessed. In detail, the obtained polymeric carriers displayed a spherical morphology with a smooth surface (Figure S2A) and showed a mono-modal narrow size distribution (polydispersity index, PDI = 0.14 ± 0.07) ranging between 150 and 400 nm with a peak at 276.4 ± 17.9 nm. Moreover, ATR-FTIR analysis qualitatively confirmed the incorporation of ICOS-Fc into the PLGA particles since the broad band around 3250 cm^−1^ ascribable to the amine A of the biomolecule, in particular to its H-bonded OH and -NH_2_ groups [50], was clearly discernible (Figure S2B). In order to quantify the amount of ICOS-Fc encapsulated into PLGA nanoparticles, a protein assay was performed on the lysis supernatant collected after the breaking out of ICOS_PLGA, resulting in an EE% of 27.2 ± 3.4% (i.e., 272.2 ± 0.3 µg). The addition of 0.5% wt of ICOS_PLGA nanocarriers to the formulation required a higher amount of liquid to extrude the paste compared with the corresponding bare formulation. Indeed, to easily inject this formulation a L/P of 0.47 was used, likely due to the tendency of PLGA to retain liquids [51].

**Figure 2:**
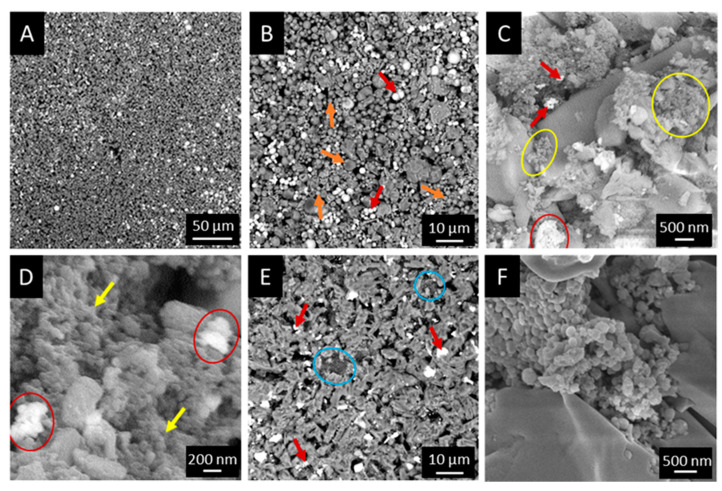
FE-SEM images of the composite cement: (**A**) Representative image to demonstrate the homogenous distribution of the powders within the CSD matrix (1000×); (**B**) Detail of the formulation with Sr-MBG-SD highlighting ZrO_2_ nanoparticles (red arrow) and Sr-MBG-SD micrometrical particles (orange arrows) (3000×); (**C**,**D**) Details of the formulation with Sr-MBG-SG highlighting the nanometric Sr-MBG-SG (yellow circles) and ZrO_2_ nanoparticles (red arrows and circle) (25,000× and 50,000×, respectively); (**E**) Details of ICOS_PLGA-containing formulation highlighting the ZrO_2_ nanoparticles (red circles) and the nanometric polymeric carries (blue circles) that fill the empty space of the CSD matrix (3000×); (**F**) Particular of ICOS_PLGA carriers in the CSD matrix (100,000×).

After the identification of the optimal L/P, the distribution of the different phases within the resorbable matrix was evaluated by means of scanning microscopy in backscattering mode. As can be observed in Figure 2A, which reports a representative image of the composite cement, it was possible to clearly identify the ZrO_2_ nanoparticles since they resulted whiter than the other cement components. ZrO_2_ nanoparticles resulted homogenously distributed throughout the matrix. The detection of both Sr-MBG and ICOS_PLGA particles was more complicated at low magnification (1000×) due to a lower atomic weight than zirconium and a smaller size. In the images acquired at 3000× and reported in Figure 2B are highlighted the ZrO_2_ particles (red arrows) and the Sr-MBG-SD particles (orange arrows), which appear darker than the radiopaque particles. On the other hand, images acquired at even higher magnifications allowed to distinguish Sr-MBG-SG (yellow arrows in Figure 2C,D, 25,000× and 50,000×, respectively) from the CSD matrix, which appeared homogenously distributed throughout the material. Concerning the microstructure of the developed cements, all the formulations with Sr-MBG-SD exhibited a disconnected matrix and porosities that were microscale in size (Figure S3A–F). In particular, the 75CSH/20 Sr-MB-SD/5ZrO_2_ (Figure S3A,B) and the 80CSH/15Sr-MBG-SD/5ZrO_2_ formulations (Figure S3C,D) showed large pores, likely due to a high amount of micro-sized bioactive particles that impaired the growth of CSD crystals. It can be ascertained that by decreasing the Sr-MBG-SD content, the uniformity of the CSD matrix increased. Indeed, the best result in terms of CSD interconnection and decreased porosity was observed for the 90CSH/5Sr-MBG-SD/5ZrO_2_ formulation (Figure 2B), whose more connected microstructure can explain the greater cohesion exhibited by the latter formulation upon hardening. Conversely, all Sr-MBG-SG formulations demonstrated a lesser and smaller porosity content when compared with those containing the micrometric Sr-MBG, regardless of their quantity (Figure S4). These results can justify the improved and satisfactory cohesion of the hardened materials with Sr-MBG-SG. Although they are homogenously distributed as micrometrical agglomerates within the matrix, the Sr-MBG-SG are small enough to fill the empty spaces between the CSD crystals and not largely interfere with their growth. A similar morphology was observed when ICOS_PLGA nanocarriers were introduced in the cement formulation. The polymeric nanoparticles were indeed located in the voids between CSD crystals (Figure 2E,F, blue arrows), leading to a uniform matrix.

**Figure 3:**
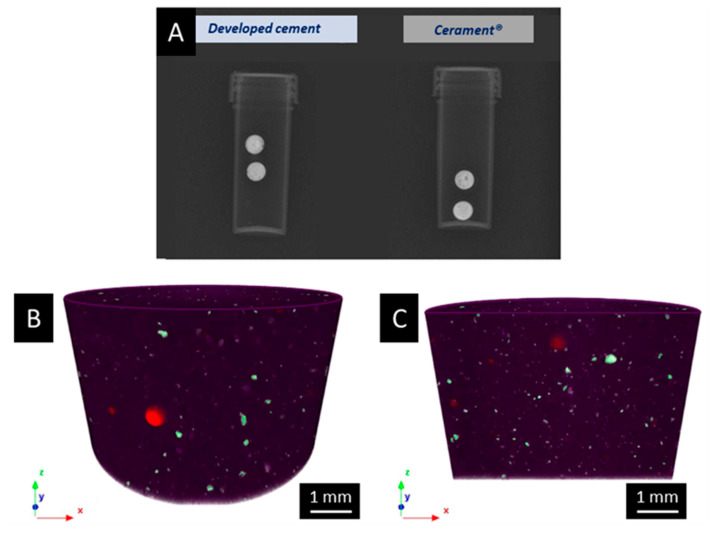
Radiopacity assessment: (**A**) Fluoroscopy imaging acquired on the developed cement (left) and commercial reference, Cerament^®^ (right); (**B**,**C**) ZrO_2_ distribution evaluation through Micro-CT analysis (purple: CSD matrix, red: air, green: ZrO_2_ particles), front view (**B**) and cross-section (**C**).

Fluoroscopy and Micro-CT analyses were completed on the selected formulations to assess the radiopacity grade, as well as the distribution of the ZrO_2_ nanoparticles throughout the cement volume. The level of radiopacity achieved by using 5% vol. of ZrO_2_ nanoparticles was sufficient for the clinical application of interest as it allowed to provide a radiopacity comparable to the commercial reference, Cerament^®^ (Figure 3A). Moreover, CTVox images showed that radiopaque nanoparticles were uniformly distributed on the edge of the sample, as well as throughout the analyzed volume (Figure 3B,C, respectively), where the ZrO_2_ material is depicted in green and the CSD matrix in purple (pores were colored in red). A different distribution was observed for Cerament^®^ for which the radiopacity was present only on the wall of the samples (Figure S5).

### 3.2. Setting Times Evaluation

The time periods when the cement can be injected (initial setting time) and after which it is completely hardened (final setting time) of the selected formulations were evaluated according to the international standard ASTM C266-04, which predicts the use of the Gillmore apparatus. Generally, the cement formulations with the nanometric Sr-MBG exhibited an initial setting time of approximately 30 min and a final setting time of 60 min (Table 3), time periods that are comparable with those found in the literature for composite CSH-based cements [52].

Conversely, the formulation with 5% vol. of Sr-MBG-SD demonstrated setting times approximately 30 min longer than those with Sr-MBG-SG, presumably due to the micrometric dimensions of Sr-MBG-SD, which hindered and slowed down the formation of the CSD network. The presence of ICOS_PLGA nanoparticles also caused an increase of approximately 30 min compared to formulation with Sr-MBG-SG, supposedly due to the higher liquid amount and to their different textural properties. Moreover, the addition of a further component might slow down the growth of CSD crystals.

Due to the injectability, qualitative mechanical properties and setting time suitable for clinical applications, as well as considerations on the incorporated amount of bioactive and pro-osteogenic particles among the different formulations without ICOS-Fc, the 75CSH/20Sr-MBG-SG/5ZrO_2_ and 90CSH/5Sr-MBG-SD/5ZrO_2_ ones have been selected as the best promising ones and have been further investigated and compared. Although the formulation with Sr-MBG-SD showed the longer setting times among all the tested ones, for the sake of comparison, the investigation was carried out both in formulation based on micrometric (Sr-MBG-SD) and nanometric MBG (Sr-MBG-SG) in order to highlight the effect that MBG particles with different properties (SSA and particularly the size dimension) could have on mechanical properties and degradation behavior of the composite cements. Moreover, although the calculated setting times are much higher compared to the commercial reference Cerament^®^, a prolonged injection period (also known as injection period) can be advantageous from a practical point of view since it could allow the surgeon to treat multiple vertebral bodies starting from the same prepared paste and these possibilities will allow to better align with the healthcare provider’s requests. On the other hand, different strategies are reported in the literature for potentially accelerating the hardening of CSH-based cements (i.e., the final setting time) without changing their formulation. Hence, a further improvement of the presented formulations could involve the addition of acids as succinic acid or citric acids, or the use of a salt solution [53,54] in the liquid phase, or CSD crystals in the powder phase [55,56].

### 3.3. Mechanical Properties Evaluation under Compression

The compressive strength of cements used for vertebral fractures is a crucial aspect considering their key function of providing mechanical support and stabilizing the fractured vertebral bodies. In particular, the compressive strength of a vertebral cement should match that of healthy human vertebral bodies (i.e., ranging from 2 to12 MPa [19,57]) to avoid the effect of “pillaring”, and thus, ensure the safety and durability of the material. The mechanical properties of the best cement formulation were measured under compressive loading in accordance with the standard ISO 5833-2002.

**Figure 4:**
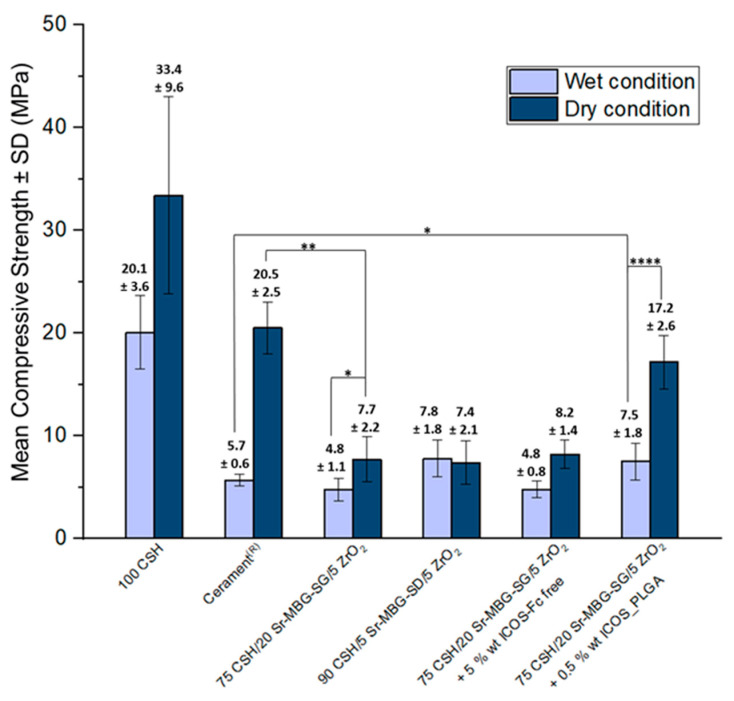
Compressive strength values of the investigated formulations measured in accordance with the ISO 5833-2022 standard under wet and dry conditions (i.e., after 24 h and 7 days of setting at 37 °C, respectively). (* *p* < 0.05, ** *p* < 0.01, **** *p* < 0.0001).

From the compressive strength data (Figure 4) it can be observed that the presence of dispersed Sr-MBG particles resulted in a decrease in the compressive strength compared to the controls, regardless of the particle size and the test conditions. However, the measured values were within the range for the compressive strength of trabecular bone tissue [19,57]. Therefore, both cement formulations could be suitable for implantation within the vertebral body. The presence of two different dispersed particles in the CSD matrix, Sr-MBG and ZrO_2_, resulted in lower compressive strength values when compared to both controls. It is postulated that this finding is due to the interruption that Sr-MBG particles caused to the overall interconnectivity of the CSD matrix, regardless of their dimension [58,59].

The introduction of the polymeric ICOS-Fc carriers significantly improved the compressive strength, achieving 17.2 ± 2.6 MPa in dry conditions (Figure 4). Furthermore, the formulation with the ICOS_PLGA particles demonstrated a greater resistance to compressive loading compared to Cerament^®^ when set for 24 h at 37 °C and a similar compressive strength when tested under dry conditions (5.7 ± 0.6Mpa and 20.5 ± 25 Mpa under wet and dry conditions). This result could be due to the reinforcing effect of the PLGA nanoparticles within the CSD matrix and their ability to retard crack propagation through the cement [60,61].

To correlate the degree of porosity to the mechanical properties of the cement, a 3D analysis on different volumes of the samples was conducted by means of the CTAn software. The percentage of porosity and the percentage of pores with a diameter lower than 300 µm using three different ranges are reported in Table 4. Data show that when Sr-MBG particles were dispersed in the matrix, the porosity increased compared to controls, reaching values of 9.1 ± 0.6%. Even higher values were detected for the formulation with ICOS_PLGA for which the porosity was estimated at 24.9 ± 1.4%. Although this formulation exhibited the highest porosity compared to the other cement studied, most of the pores had a diameter lower than 50 µm or 100 µm (Table 4) and only 1.6 ± 0.8% were greater than 500 µm (Figure S6). Moreover, a qualitative representation of the pore distribution throughout the cement volumes is reported in Figure S7. This result, together with the one that was mentioned for the SEM observations (Figure 2E,F), can justify the superior mechanical performance of the ICOS_PLGA-containing formulation. Generally, both controls and the developed formulations showed at least 90% of pores with diameters lower than 300 µm. Indeed, an inability in the matrix’s cohesion occurred when the majority of pores demonstrated a diameter greater than 300 µm, as observed for the 75CSH/20Sr-MBG-SD/5ZrO_2_ formulation (Figure S3G).

### 3.4. In Vitro Degradation Rate and Bioactivy Behaviour

The degradability of CSH-based cements is one of the key features of their clinical application. For this reason, appropriate in vitro resorption tests were completed on the best formulations and on the control formulations (e.g., 100CSH and Cerament^®^) in Tris-HCl according to standard ISO 10993-14. Although from a macroscopic perspective, the friability of 90CSH/5Sr-MBG-SD/5ZrO_2_ was significantly reduced compared with the other tested formulations with micrometric MBGs, the sample showed an insufficient cohesion upon contact with the buffer solution. This behavior will result harmful from a clinical point of view since it will lead to a reduced ability to provide mechanical support. Accordingly, the 90CSH/5Sr-MBG-SD/5ZrO_2_ formulation was considered unsuitable for applications in vertebroplasty, and thus, discarded from further examinations. Therefore, the optimal formulation of the developed injectable cement was the 75CSH/20Sr-MBG-SG/5ZrO_2_.

**Figure 5:**
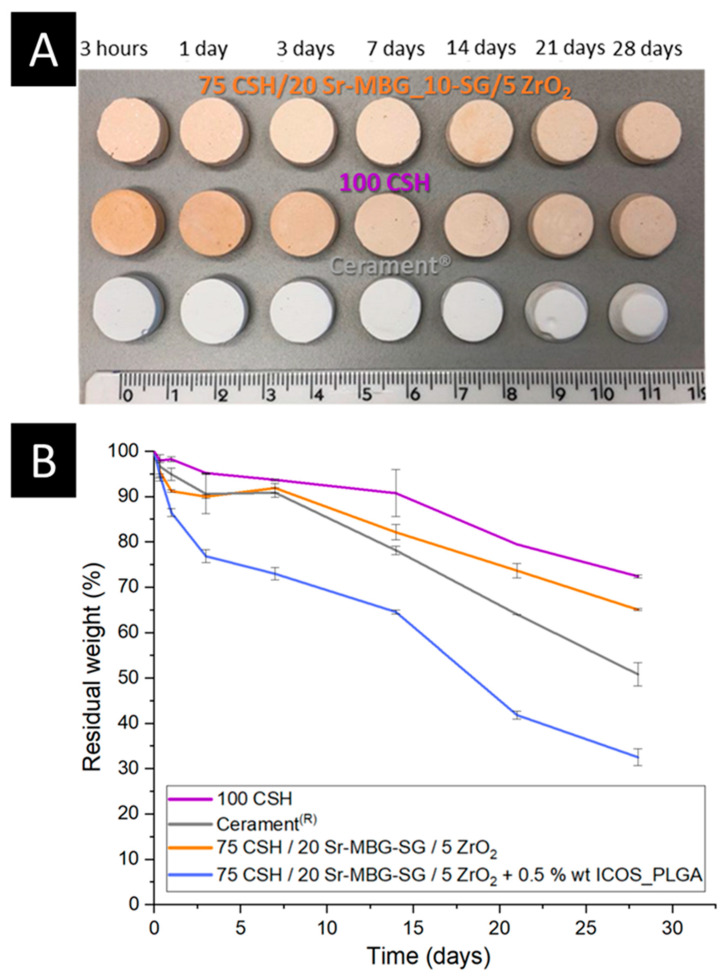
Degradation kinetics of tested samples that degrade by bioerosion process (**A**) and weight loss of samples once immersed in Tris-HCl up to 28 days (**B**).

Figure 5A reports the resorption kinetics of the different CSH-based cements under investigation and it can be noted that the degradation of the samples occurred from their surface. It is, therefore, a surface erosion process that led to a gradual decrease in the dimension of the samples over the experimental period, typical of the degradation mechanism exhibited by CSH-based cements [52,62,63]. The degradation kinetics were also evaluated by measuring the percentage of residual weight at each timepoint and the obtained profiles are reported in Figure 5B. Results indicated that after 28 days of soaking the optimized cement displayed a residual mass percentage of 65.0 ± 0.2%, an intermediate value between Cerament^®^ (40.3 ± 0.5%) and 100CSH (72.5 ± 0.4%). In contrast, the introduction of the polymeric ICOS-Fc nanocarriers led to an acceleration in the rate of cement degradation, even faster than Cerament^®^. Indeed, an abrupt mass loss compared to the pristine formulation was calculated, reaching residual weight values of 32.5 ± 1.8% after 28 days, supposedly due to the greater microporosity of the formulation with ICOS_PLGA (Table 4) that allowed water diffusion in the inner part of the sample. The complete dissolution of the sample was recorded at 85 days, 42 days, 96 days and 56 days for the composite cement without and with ICOS_PLGA, 100CSH and Cerament^®^, respectively.

Moreover, the developed cements demonstrated a greater weight loss on the first day of immersion in Tris-HCl compared to the controls (Figure 5B), which can be linked to a significant released amount of Ca^2+^ ions. It is known that the release of a large amount of ions into the body environment is detrimental to cell viability and, in turn, results in toxicity of the biomaterial [64,65]. Based on this consideration, the concentration of Ca^2+^ ions released over time in the buffer was assessed using ICP-AES. Results demonstrated a released Ca^2+^ concentration of about 130 ppm and 200 ppm of Ca^2+^ in the first day of observation for the cement without and with the ICOS-Fc carrier, respectively (Figure S8). Moreover, a preliminary ongoing cellular experiment with MC3T3-E1 cell line demonstrated the full biocompatibility of the developed biomaterials.

**Figure 6:**
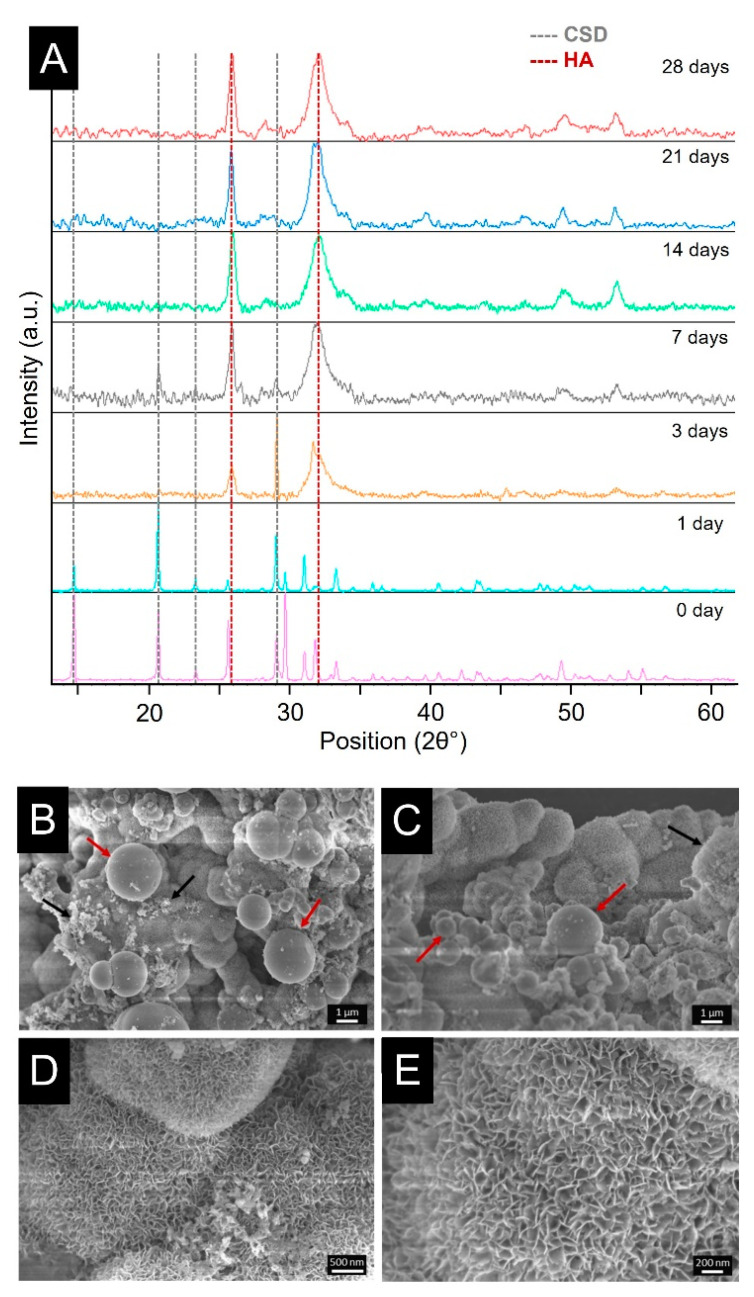
Bioactive behavior assessment of 75CSH/20Sr-MBG-SG/5ZrO_2_ formulation: (**A**) XRD spectra of delaminated sheets acquired at different immersion times (CSD peaks in grey dotted lines, HA peaks in red ones); (**B**–**E**) FE-SEM images captured after 28 days of the immersion in SBF; (**B**,**C**) Agglomerates of newly formed HA (black arrows) and ZrO_2_ particles (red arrows) are highlighted (10,000×); (**D**,**E**) Crystalline HA observed at higher magnification (50,000× and 100,000×, respectively).

After the investigation of the degradation rate, the ability of the optimized cements to induce the precipitation on their surface of a layer of HA with similar morphology and composition to the one present in bone tissue has been investigated. During testing, a delamination phenomenon at the surface occurred, and thus, confirmed that CSH-based cement degraded by means of a bioerosion process. The XRD spectra (Figure 6A) of the optimized formulation highlights the peaks related to CSD and HA [66]. Intense peaks at 25.9° and 31.8° related to crystalline HA were clearly visible already on the delaminated sheets collected after 3 days. Indeed, XRD spectra after 7 days were dominated by HA peaks, demonstrating the full coverage of the sheets with a crystalline apatite-layer. Alongside, FE-SEM observations demonstrated the typical morphology of the HA layer on the delaminated samples collected from the developed composite cement. In particular, a layer of crystalline HA covered the whole analyzed area after 21 days of immersion in SBF, presenting the typical cauliflower morphology along with the presence of newly formed non-crystalline aggregates (Figure 6B,C, black arrows). Furthermore, the ZrO_2_ particles can be easily recognizable since they maintain a smooth surface (Figure 6B,C, red arrows), while the Sr-MBG-SG particles were no longer visible as they were completely covered by HA. At higher magnifications, it was possible to better appreciate the morphology of crystalline HA and the leaf-like needle structure (Figure 6D,E). The bioactivity of the developed biomaterial is also improved due to the presence of a large amount of silanols group (Si-OH) at the surface of Sr-MBG, allowing a very fast ion-exchange reaction and protein adsorption once the cement is implanted, greatly promoting its osteo-integration [67].

At each tested time point, the pH of the collected supernatants was measured to evaluate if the values were suitable for the cellular physiological activity [46] and no significant differences were found with respect to the initial SBF pH (pH = 7.41 ± 0.8).

### 3.5. Sr^2+^ and ICOS-Fc Release Kinetics from the Optimized Cement

One of the innovative aspects of the developed cement is the presence of 10 mol % of pro-osteogenic strontium ion in the MBG particles enclosed within the bioresorbable matrix and the other one is the introduction of the recombinant protein ICOS-Fc to impart pro-osteogenic and anti-osteoclastogenic features, respectively. Release tests were, therefore, performed to evaluate the release profile of these two stimuli.

**Figure 7:**
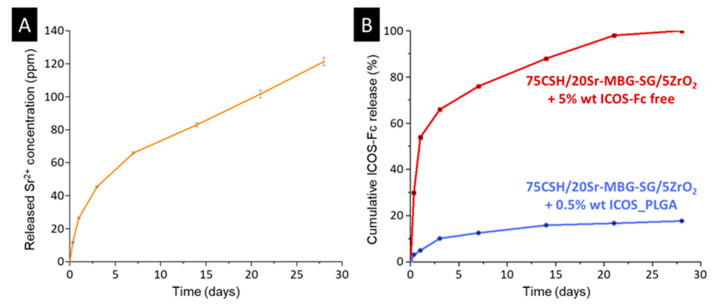
Strontium ion release kinetics from the optimized composite cement formulation (**A**) and ICOS-Fc release profile from the anti-osteoclastogenic cements (**B**) with free dispersed ICOS-Fc (red line) and the formulation containing ICOS_PLGA (blue line).

As reported in Figure 7A, the CSD matrix of the optimized cement was able to modulate the strontium burst release observed after 3 h of immersion characteristic of the nanometric Sr-MBG-SG particles as such [44], providing a sustained release of the therapeutic ion. By comparing the maximum concentration of strontium reached over time with the concentrations resulting from acid attacks (registered at 175.0 ± 17.3 ppm), it can be deduced that after 28 days of immersion in Tris-HCl, a residual amount of strontium ion was still inside the cement. In fact, the highest ion concentration, measured in correspondence of the 28 days of immersion, was equal to 120.2 ± 5.4 ppm, while the maximum achievable release was of about 175 ppm.

As far as the anti-osteoclastogenic biomolecule is concerned, the release kinetics obtained from cement formulation with 5% wt of ICOS-Fc directly dispersed showed a burst release in the first 24 h of observation (Figure 7B, red line): 29.73 ± 0.05% of the biomolecule was released already after 1 day of immersion. The initial amount of introduced ICOS-Fc was completely released after 28 days exhibiting a suboptimal profile. On the other hand, the use of polymeric delivery carriers as ICOS_PLGA nanoparticles significantly reduced the initial release (Figure 7B, blue line) and allowed a prolonged therapeutic effect of ICOS-Fc with regard to the direct dispersion of the biomolecule and the ICOS_PLGA alone (Figure S2C). Indeed, only 17.86 ± 0.12% of the incorporated amount of the biomolecule was released by the anti-osteoclastogenic formulation after 28 days. This result indicated that the 75CSH/20Sr-MBG-SG/5ZrO_2_ + 0.5% wt ICOS_PLGA formulation was able to offer a prolonged anti-osteoclastogenic stimulus, ideally up to the complete dissolution of the cement.

## 4. Discussion

In the present research, the effects of two different types of Sr-MBG particles on the properties of the final cement were investigated with the intention of developing the optimal cement formulation in terms of injectability, setting times, mechanical properties, as well as the resorption rate for the treatment of osteoporotic vertebral compression fractures.

The attention was firstly focused on the identification of the appropriate L/P to obtain a cement paste with satisfactory flowability through a 13 Gauge needle in terms of consistency and injectability. The optimization of this parameter is a fundamental step in the development of cement since it directly affects many properties of the material, such as injectability and setting times, besides mechanical strength [48,49]. Generally, from the obtained results it can be stated that by increasing Sr-MBG content, a higher amount of liquid is required to obtain an injectable paste. This behavior can be related to the high textural properties of both used Sr-MBGs, in particular, their SSA and pore volume that led to the adsorption of a larger amount of liquid phase. In addition, the high density of hydrophilic silanol groups (Si-OH) on their surface implied that these particles can easily interact with the aqueous liquid phase, thus, partially subtracting it from the solubilization/hydration reaction of CSH. Based on this consideration, higher L/P values should be used for the formulations with the Sr-MBG-SG compared to the Sr-MBG-SD since they possess a higher SSA [44], and consequently, a greater surface responsiveness with the liquid, leading to higher water demand. However, comparing the same volume formulations, it can be noted that the L/Ps of the formulations containing Sr-MBG-SD are higher than those containing Sr-MBG-SG. This result can be attributable to the morphological properties of Sr-MBG-SD that present micrometric dimensions with a rather large dimensional distribution, which affects their compactability, also named “packing density” [68]. In addition, the lower SSA of Sr-MBG-SD along with their irregular and bigger dimensions had adverse effects on the injectability of the formulations and the consistency of hardened samples, respectively.

Other key factors for the clinical applications in VP of injectable cements are the setting properties defined as the time periods when the cement can be injected (initial setting time) and when it is completely hardened (final setting time). Specifically, an ideal cement should present setting times that allow an easy injection procedure at OR temperature (e.g., initial setting time of approximately 15–25 min) and a subsequently rapid hardening within the vertebral body (e.g., final setting time of approximately 60 min) to quickly move the patient out of the OR. From results obtained following the standard ASTM C-266-04, we demonstrated that all the investigated formulations provided timeframes ranging in the desired interval of operation, and thus, were suitable for clinical use. In particular, the cements based on nanometric Sr-MBG resulted in an initial setting time of approximately 30 min and a final setting time of approximately 60 min, intervals that are comparable with those found in the literature for composite CSH-based cements [52]. Focusing the attention on the different investigated formulations, it can be ascertained that as the Sr-MBG-SG content increases, both setting times increase. This observation can be due to the increase in the L/P value and has also been reported by other authors in the literature [52,59]. In fact, the amount of water needed to inject the composite paste is in part used for the CSH hydration and the remaining water will evaporate during the setting phase; the higher the water content to ensure the injection of the paste, the longer the time required for the residual water evaporation will be. On the other hand, the formulation with 5% vol. of Sr-MBG-SD showed setting times of about 30 min longer than those with Sr-MBG-SG, presumably due to the micrometric dimensions of the involved pro-osteogenic particles, which hindered and slowed down the formation of the CSD network. Although the developed cements were characterized by longer final setting times compared to the commercial reference Cerament^®^, the calculated injection intervals represented a very promising feature for the potential marketing of these materials. A slightly longer injection interval could offer the clinician the time to stabilize multiple fractures within the vertebra using a single package of cement.

At this stage, to define the most promising formulation of the cement, the 75CSH/20Sr-MBG-SG/5ZrO_2_ and 90CSH/5Sr-MBG-SD/5ZrO_2_ formulations were selected and further investigated due to their injectability, qualitative mechanical properties, setting time suitable for clinical applications and considerations on the incorporated amount of bioactive and pro-osteogenic particles.

Prior to the evaluation of the mechanical resistance of the selected cements, their radiopacity was assessed, since it is an essential property of injectable cement for VP application, for the purpose of visualizing, under fluoroscopy, the biomaterial during its injection inside the fractured vertebral body to detect possible extra-vertebral leakages early and to facilitate the follow-up. The level of radiopacity achieved using the introduction of 5% vol. of ZrO_2_ nanoparticles in the formulation provided a radiopacity comparable to the commercial reference as assessed by fluoroscopy imaging, and thus resulted sufficient for the clinical application of interest. Moreover, the developed formulations demonstrated a good distribution of the radiopaque particles throughout the analyzed volume. The outcomes suggested that the mixing methods adopted to homogenize the different powders, as well as the manual mixing of the powder and liquid phases led to a uniform paste with homogenous distribution of the radiopaque phase. In contrast, Cerament^®^ exhibited radiopacity only at the outer surface of the samples. This behavior is due to the different strategy used by the supplier to introduce the radiopaque element in the formulation of the cement compared to the one adopted in our work; since the radiopaque element is dispersed in the liquid phase of Cerament^®^, the diffusion and subsequent evaporation of water during the sample-setting phase probably led to a higher concentration of iohexol close to the outer surface. Irrespective of the type of matrix (i.e., polymeric and inert or calcium-based and resorbable), injectable cements currently on the market can be classified into two categories based on the approach used to impart radiopacity: the ones that foresee the employment of a liquid radiopaque phase, such as Cerament^®^, and those that used particles ZrO_2_ or barium sulphate (BaSO_4_), such as SmartSet™ HV Bone Cement (DePuy, Warsaw, USA) and SmartSet™ Endurance Bone Cement MV (DePuy, Warsaw, USA) or Simplex^®^ P Radiopaque Bone Cement (Stryker, Kalamazoo, MI, USA), respectively [69,70]. Both routes are efficacious; however, the use of a radiopaque liquid can result in an inhomogeneous distribution of the radiopaque element and can cause a burst release at the site of injection, thus preventing an effective follow-up. Conversely, the radiopacity of the developed composite cements will be guaranteed by the presence of the ZrO_2_ particles incorporated within the matrix and, in this case, the visualization of the cement during the follow-up will be possible also after the full resorption of the cement as the particles will remain encased within the new bone matrix.

Besides all the discussed features and considering its mechanical support role in the fractured vertebral bodies, the developed cement must demonstrate appropriate mechanical properties to allow the load distribution across the adjacent vertebrae, preventing new fractures and avoiding the effects of “pillaring”. Adequate mechanical properties also ensure safety and durability, as well as increase the performance of the vertebroplasty implant. In particular, the mechanical characteristics of a successful cement for vertebral applications should match those of the host tissue to reduce the chance of complications such as post-operation stress shielding, implant-related osteopenia or subsequent refracture [71]. In this context, cements should present a compressive strength of 2–12 MPa and an elastic modulus of 0.1–5 GPa, values that are characteristic of healthy human vertebral cancellous bone [19,57]. Based on results measured in accordance with the standard ISO 5833 acquired under both wet and dry conditions, the presence of dispersed particles within the CSD matrix led to a decrease in the compressive strength with respect to the controls, regardless of the particle size and the test conditions. This finding is due to the interruption that MBGs caused to the CSD matrix interconnectivity, regardless of their dimension. Besides the formulation of the powder phase, the total amount of porosity and the pore size strongly influence the mechanical properties of cements. Indeed, Cerament^®^ and 100CSH demonstrated the lowest porosity levels and in turn exhibited the higher compressive strength values. In contrast, the formulations containing Sr-MBGs and ZrO_2_ exhibited an increase in the porosity content, together with a reduction in the mechanical properties, for both dry and wet conditions. This is an expected behavior since the presence of various particles in the powder phases characterized by a different granulometry could increase the amount of incorporated air during the mixing phase, and consequently, the formation of pores during the injection and setting phases. However, the most promising cements, the 75CSH/20Sr-MBG-SG/5ZrO_2_ and the 90CSH/5Sr-MBG-SD/5ZrO_2_, demonstrated a compressive strength of approximately 4–7 MPa, which is within the characteristic range of trabecular bone tissue [19,57]. Therefore, it can be stated that the developed formulations can be suitable for implantation within the fractured vertebral body.

Nevertheless, the developed CSH-based cements possessed the fundamental properties of resorbability once in contact with the body, essential for promoting tissue response. This feature is known to improve the interaction of the biomaterial with the physiological environment and to promote cellular colonization of the implant and consecutive osteointegration and tissue regeneration [46,72]. However, resorbable biomaterials should possess degradation kinetics complementary to bone regeneration timing in order to be used in clinics. It is generally accepted that bioresorbable materials with a degradation rate faster than that of bone regeneration are not suitable because they do not provide adequate long-term mechanical support [17,73]. For this reason, appropriate in vitro resorption tests were completed in accordance with standard ISO 10993-14, and the degradation rates, expressed in terms of residual weight of the developed cements, were compared with those of controls 100 CSH and Cerament^®^. Notably, when cylindrical samples of 90CSH/5Sr-MBG-SD/5ZrO_2_ formulation were immersed in Tris-HCl buffer, they did not show adequate cohesion, although from a macroscopic point of view, their friability was significantly reduced compared with the other tested formulations containing Sr-MBG-SD. This behavior can be attributed to the poor entanglement of CSD crystals due to the presence of micrometrical particles and to the presence of large pores, which leads to the collapse of the sample once in contact with the liquid. Such occurrence in clinics could lead to serious consequences for the patients since a poorly cohesive cement, once in contact with physiological fluids, would have a reduced ability to provide mechanical support. Therefore, the formulation with Sr-MBG-SD was considered unsuitable for its applications in vertebroplasty, and thus, discarded from further examinations. On the contrary, the formulation with Sr-MBG-SG exhibited excellent cohesion degradation features when in contact with the degradation buffer. Accordingly, degradation tests and further investigations were conducted on the optimal developed formulation: the 75CSH/20Sr-MBG-SG/5ZrO_2_. This formulation showed suitable degradation kinetics with a rate intermediate compared to those of controls that could better complement the timing of bone tissue regeneration. In particular, the increase in degradation kinetics compared to 100CSH is due to the lower content of CSD in the developed cement. Moreover, the presence of Sr-MBG-SG and ZrO_2_ particles within the matrix led to a higher porosity that allowed a higher buffer permeability, which resulted in an acceleration of the solubilization of the CSD matrix. In view of developing an innovative resorbable cement for VP applications, the obtained degradation kinetics that were longer than the commercial reference can represent an important advantage since it is largely reported in the literature that a common drawback of CSH-based cements is related to their too rapid in vivo degradation rate compared with the bone tissue regeneration, which can lead to a momentary lack in mechanical support [74,75].

Alongside the degradation test and considering that a key feature of the developed cement is the presence of a significant amount of pro-osteogenic strontium ions in the MBG particles, the strontium release profile was evaluated. The optimized cement showed a sustained release of strontium ions over 28 days, characterized by a gradual progression over time. This kinetics is a great advantage since the well-known therapeutic effect of strontium of stimulating OB differentiation and their activity of new extracellular matrix deposition while inhibiting the OC differentiation and resorption [25] can be prolonged favoring the long-term tissue regeneration process. In particular, the released strontium ions take part in the RANK/RANKL/OPG signaling pathway [24,25,26], fundamental path of the bone remodeling process. Some research studies have suggested that Sr^2+^ ions can increase the secretion of OPG and inhibit that of RANKL by OBs interacting with the CaSR receptors on their cell membranes, resulting in a stimulation of OBs activity [27]. Cellular in vitro assessments are currently ongoing to assess this aspect provided by the developed biomaterial; however, the pro-osteogenicity of the strontium ions released by MBGs has been previously reported [44,76]. Moreover, the optimized biomaterial can be a valid and effective alternative to the current standard pharmaceutical treatment for OP that foresees the assumption of a strontium ranelate-based drug [22]. The assumption of strontium ranelate was reduced over the last years due to the negative side effects, especially venous thromboembolic events and DRESS syndrome [77]. The developed cement can provide a localized and sustained release of pro-osteogenic strontium ions directly injected into the bone defect, avoiding a systemic administration of drugs.

The optimized cement presented a clear bioactive behavior after 1 day of contact with SBF due to the presence of MBG and the use of calcium-based matrix. The ability of cements consisting exclusively of CSH to induce the precipitation of HA on their surface has been reported in the literature [15,16,78,79]. The presence of this mineral apatite phase makes the cement surface particularly osteoconductive and suitable for colonization of bone tissue cells, which will be able to continue the physiological process of remodeling. Moreover, Ricci et al. and Walsh et al. hypothesized that the presence of sulphate ions operates on cell signaling pathways [16]. These ions, alongside other signaling proteins such as growth factors released after the OC resorption activity, act on the gene expression of OBs, promoting their maturation and activity [16]. The high amount of calcium ions also represents a stimulus for OB activation and, at the same time, tends to inhibit OC activity [80]. On the other hand, during the bioactivity test, a delamination phenomenon was observed, probably caused by the crystallization of HA on the surface of the samples. Once the HA deposited by ion exchange between the cement and the SBF began to crystallize, it lost the bond with the bulk sample and detached itself in the form of crystalline sheets. This interpretation would also explain the presence of non-crystalline HA deposits on the surface of the bulk samples. The detachment of these superficial sheets from the samples along with the bioerosion resorption process allowed the immediate exposition of the underneath surface to ion exchange reactions, which in turn led to the progressive deposition of new agglomerates of HA. This process continued until the complete dissolution of the samples occurred. Presumably, a similar phenomenon can recur also in vivo, and, in this way, the composite cement developed in our work could support bone regeneration throughout its resorption since the continuous formation of HA can constitute a powerful steady osteoproductive stimulus.

Considering the osteoporotic scenario in which vertebral body fractures mainly occur, the main innovation of the present work was the introduction of the biomolecule ICOS-Fc in the optimized cement formulation with the aim of providing anti-osteoclastogenic feature. Therefore, the composite cement will synergistically exploit the bioactive behavior of MBGs, the pro-osteogenic effects of released strontium, as well as the biological stimulation due to the presence of ICOS-Fc. For this purpose, ICOS-Fc was incorporated into the 75CSH/20Sr-MBG-SG/5ZrO_2_ formulation exploiting different routes: its direct dispersion within the liquid phase or through the addition of polymeric PLGA nanoparticles containing ICOS-Fc (i.e., ICOS_PLGA). Both anti-osteoclastogenic formulations were fully characterized and the different biomolecule release kinetics were investigated.

Generally, as expected, the addition of ICOS-Fc in its free form did not affect the characteristics of the cement. Indeed, the dispersion of free ICOS-Fc into the liquid phase did not involve any change in the L/P value and injectability of the pristine formulation, which remained equal to 0.40 and 100%, respectively, as calculated for the optimized cement. This behavior was also exhibited for other cements in which free drugs were dispersed within the matrix, such as Cerament^®^ G [81] and Cerament^®^ V [82], that foresee antibiotic gentamicin and vancomycin antibiotic, respectively, in the traditional formulation of Cerament^®^.

However, a higher amount of liquid was required to extrude the paste when 0.5% wt ICOS_PLGA nanocarriers were added. Indeed, to easily inject this formulation a L/P of 0.47 was used, probably due to the tendency of PLGA to retain liquids and to their small size [51]. However, the paste was fully injectable after 90 s of mixing at 20 °C. The textural properties, as well as the physical presence of PLGA nanoparticles also affected the rate of hardening of the cement, causing an increase of approximately 30 min for both the calculated setting times compared with those measured for the pristine cement formulation. This phenomenon can be presumably due to the difficulty of CSD crystals to grow and interconnect as hampered by the presence of a further ingredient. In this scenario, an acceleration of the setting times could be obtained by the addition of a hardening accelerator as a CSD crystal in the starting powder phase or citric acid in the liquid phase. Although, from a clinical perspective, a prolonged injection period can be advantageous since it could allow the surgeon to treat different vertebral bodies using only one cement dose; these possibilities will allow for a better alignment with the healthcare provider’s requests. Different strategies are reported in the literature for accelerating the hardening of CSH-based cements without changing their formulation, and so a further development of the presented formulations could involve the addition of dicarboxylic acids as succinic acid or citric acids [83,84] or the use of phosphate or chloride salt solution as sodium chloride (NaCl), sodium sulphate (Na_2_SO_4_), potassium chloride (KCl), and potassium sulphate (K_2_SO_4_) [53,54] in the liquid phase or CSD crystals in the powder phase [55,56].

Interestingly, the incorporation of ICOS_PLGA nanocarriers significantly enhanced the compressive strength values in comparison to the cement as such, irrespective of the test environment conditions, achieving 17.2 ± 2.6 MPa in dry conditions. Furthermore, the ICOS_PLGA-containing formulation demonstrated higher resistance to compression with respect to Cerament^®^ when set for 24 h at 37 °C and a similar compressive strength when tested under dry conditions (5.7 ± 0.6 MPa and 20.5 ± 2.5 MPa under wet and dry conditions, respectively). This result can be ascribed to the reinforcing effect of the polymeric component within the CSD matrix due to their ability to reduce the increase in crack opening for a given displacement and the tenacity during compression followed by the delay of the complete break [85,86]. Although the development of biomaterials containing PLGA particles is currently widely used in bone tissue engineering for imparting improved and further features to the bare biomaterials [45,87,88,89], the mechanical properties of a CSC, including nanometric PLGA particles, have not been investigated to date.

As a further validation, the effects of the ICOS_PLGA particles on the degradation rate of the cements were also evaluated. From the experiments conducted by immersing dry samples in Tris-HCl buffer in accordance with ISO 10993-14, it was observed that a faster in vitro degradation rate occurred in the first week of immersion followed by a burst mass loss. This result can be attributed to the higher porosity of this formulation, leading to greater penetration of the buffer in the cement [90]. This was evident between 14 and 21 days, when a significant mass reduction occurred, indicating a significant bulk degradation of the composite material containing the polymeric carriers. An increase in porosity might be detrimental as it can lead to a decrease in the compressive strength and may conduct the failure of the implant over time [91]. However, if needed, the cement degradation rate can be tuned by optimizing the content of ICOS_PLGA nanoparticles and/or their size to better align with the regeneration rate of the bone in order to guarantee mechanical competence.

Lastly, biomolecule release tests were completed under physiological conditions to assess the ability of the final formulation to release ICOS-Fc, and thus, offer a sustained anti-osteoclastogenic stimulus. The release kinetics calculated by means of Micro BCA Protein Assay demonstrated a strong burst release in the first 24 h when the biomolecule was directly dispersed within the cement. This immediate release should not be linked to the degradation process of the sample but can indicate an accumulation of ICOS-Fc at the edges of the samples due to the evaporation process of the liquid phase during the setting at 37 °C or its diffusion within the cement matrix. A similar burst release kinetics were also observed when the biomolecule was encapsulated into the ICOS_PLGA, likely due to its localization close to the walls of the nanoparticles. Instead, once the polymeric nanocarriers were incorporated in the CSD matrix of the composite cement, a more sustained release of ICOS-Fc was observed, suggesting that the designed cement could provide a prolonged therapeutic effect. This outcome confirms that the introduction of engineered delivery systems, such as polymeric nanoparticles, can confer several advantages by providing an adequate, sustained, and localized presentation of the active biomolecule in a time-dependent manner, besides enhancing the physical characteristics of the host biomaterials.

## 5. Conclusions

In the present investigation, a novel injectable resorbable composite cement combining for the first time pro-osteogenic and anti-osteoclastogenic features useful for the stabilization and treatment of vertebral body fractures has been designed and successfully developed.

To obtain the best formulation in terms of injectability, setting times and mechanical properties, as well as resorption rates and different formulations of the cement have been tested by tuning the content of the CSH matrix and Sr-MBG. Moreover, the effects of two different types of Sr-MBG particles on material properties have been examined. Results demonstrated that cement with 20% vol. of nanometric Sr-MBG-SG dispersed within the formulation presented the best performance, especially in terms of setting times and mechanical properties. Moreover, the developed cements were able to provide a level of radiopacity comparable to the commercial reference Cerament^®^, homogenously distributed throughout the entire volume of the materials.

The innovative aspect of the present cement concerns the ability to simultaneously release, with a sustained profile, a pro-osteogenic cue (i.e., Sr^2+^ ions), crucial for bone regeneration and an anti-osteoclastogenic biomolecule (i.e., ICOS-Fc) fundamental in an OP scenario. In particular, it was observed that the incorporation of PLGA nanoparticles containing ICOS-Fc enhanced the mechanical properties of the cement beyond conferring a prolonged therapeutic effect.

To the best of the authors’ knowledge, this is the first reported study related to the optimization and development of a CSH-based resorbable cement with both anti-osteoclastogenic and osteoproductive cues that, thus, can be effective against OP, which are responsible of compression vertebral body fractures.

Overall, the results indicated that the optimized cement is promising for the treatment of vertebral body compressive fractures and could be able to stimulate an appropriate bone remodeling response, favoring an effective healing.

Cellular in vitro tests using both OBs and OCs are currently ongoing to validate the osteogenic potential of the cement and better understand its anti-osteoclastogenic behavior. In addition, future in vivo tests on small and large animals will be useful to confirm the efficacy of the developed cement.

## Figures and Tables

**Table 1:** Developed formulations of the injectable composite cement.

Formulation	*v*/*v*%
75CSH/20Sr-MBG/5ZrO_2_	75CSH/20Sr-MBG-SG/5ZrO_2_
	75CSH/20Sr-MBG-SD/5ZrO_2_
80CSH/15Sr-MBG/5ZrO_2_	80CSH/15Sr-MBG-SG/5ZrO_2_
	80CSH/15Sr-MBG-SD/5ZrO_2_
85CSH/10Sr-MBG/5ZrO_2_	85CSH/10Sr-MBG-SG/5ZrO_2_
	85CSH/10Sr-MBG-SD/5ZrO_2_
90CHS/5Sr-MBG/5ZrO_2_	90CSH/5Sr-MBG-SD/5ZrO_2_
Cerament^®^ (control)	CSH-based commercial cement (% wt 60CSH/40 nanoHA)
100 CSH (control)	

**Table 2:** Optimized L/P and corresponding injectability for the tested formulations of the injectable cement.

Formulation (*v*/*v*%)	Optimized L/P	Injectability (%)
75CSH/20Sr-MBG-SG/5ZrO_2_	0.40	~100
75CSH/20Sr-MBG-SD/5ZrO_2_	0.45	~75
80CSH/15Sr-MBG-SG/5ZrO_2_	0.35	~100
80CSH/15Sr-MBG-SD/5ZrO_2_	0.40	~75
85CSH/10Sr-MBG-SG/5ZrO_2_	0.30	~100
85CSH/10Sr-MBG-SD/5ZrO_2_	0.35	~100
90CSH/5Sr-MBG-SD/5ZrO_2_	0.27	~95
75CSH/20Sr-MBG-SG/5ZrO2 +5% wt ICOS-Fc free	0.40	~100
75CSH/20Sr-MBG-SG/5ZrO2 +0.5% wt ICOS_PLGA	0.47	~100
Cerament^®^	0.40	~80
100 CSH	0.20	~100

**Table 3:** Setting times of the selected cement formulations and controls evaluated in accordance with the standard ASTM-C266-04.

Formulation (*v*/*v*%)	Optimized L/P	Initial Setting Time (Min)	Final Setting Time (Min)
75CSH/20Sr-MBG-SG/5ZrO_2_	0.40	35 ± 2	64 ± 2
80CSH/15Sr-MBG-SG/5ZrO_2_	0.35	33 ± 2	62 ± 2
85CSH/10Sr-MBG-SG/5ZrO_2_	0.30	19 ± 3	57 ± 1
90CSH/5Sr-MBG-SD/5ZrO_2_	0.27	49 ± 1	92 ± 2
75CSH/20Sr-MBG-SG/5ZrO2 +5% wt ICOS-Fc free	0.40	32 ± 4	66 ± 2
75CSH/20Sr-MBG-SG/5ZrO2 +0.5% wt ICOS_PLGA	0.47	56 ± 2	96 ± 6
Cerament^®^	0.40	12 ± 4	39 ± 3
100 CSH	0.20	7 ± 2	18 ± 3

**Table 4:** Percentage of porosity and percentage of the pores of the investigated cements calculated by means of CTAn software with diameter lower than 50 µm, between 50 and 100 µm and between 100 and 300 µm.

Formulation (v/v%)	Porosity (%)	Pores ≤ 50µm (%)	Pores 50–100 µm (%)	Pores 100–300 µm (%)
75CSH/20Sr-MBG-SG/5ZrO_2_	9.1 ± 0.6	22.1 ± 3.8	33.7 ± 1.2	41.3 ± 5.9
90CSH/5Sr-MBG-SD/5ZrO_2_	8.8 ± 1.8	22.5 ± 5.4	22.0 ± 6.4	51.3 ± 8.9
75CSH/20Sr-MBG-SG/5ZrO_2_ +5% wt ICOS-Fc free	10.8 ± 0.2	26.4 ± 4.1	13.9 ± 0.3	51.9 ± 7.4
75CSH/20Sr-MBG-SG/5ZrO_2_ +0.5% wt ICOS_PLGA	24.9 ± 1.4	39.9 ± 6.8	27.5 ± 1.4	26.7 ± 4.9
Cerament^®^	2.0 ± 0.5	35.5 ± 8.5	28.9 ± 3.5	33.9 ± 5.3
100 CSH	0.8 ± 0.1	75.9 ± 2.1	3.2 ± 0.6	19.9 ± 1.9

## Data Availability

The data presented in this study are openly available in ZENODO at the following dois: 10.5281/zenodo.7497228; 10.5281/zenodo.7497224; 10.5281/zenodo.7497236; 10.5281/zenodo.7497250.

## Supplementary Materials

The following are available online at https://www.mdpi.com/article/10.3390/biom13010094/s1. The original contributions presented in the study are publicly available. Figure S1. Characteristics and morphology of the commercially available ZrO2 nanoparticles exploited to impart radiopacity to the cement as well as of the two types of Sr-MBGs separately included within the formulation to impart bioactive and pro-osteogenic features. Figure S2. ICOS_PLGA nanoparticles characterization: (A) FE-SEM images of ICOS_PLGA nanoparticles (25,000×). (B) ATR-FTIR spectra of ICOS-Fc (black line), unloaded PLGA nanoparticles (red line) and ICOS_PLGA carriers (green line). (C) Cumulative amount of released ICOS-Fc from PLGA nanoparticles as a function of time determined by means of Micro BCA Protein Assay. Figure S3. SEM images of cement formulations containing Sr-MBG-SD acquired at 1000× and 3000×: (A,B) 75CSH/20Sr-MBG-SD/5ZrO2, (C,D) 80CSH/15Sr-MBG-SD/5ZrO2, (E-F) 85CSH/10Sr-MBG-SD/5ZrO2. (G) Pore size distribution of the 75CSH/20Sr-MBG-SD/5ZrO2 formulation. Figure S4. SEM images of cement formulations containing Sr-MBG-SG acquired at 1000× and 3000×: (A,B) 75CSH/20Sr-MBG-SG/5ZrO2, (C,D) 80CSH/15Sr-MBG-SG/5ZrO2 and (E,F) 85CSH/10Sr-MBG-SG/5ZrO2. Figure S5. Radiopacity distribution of Cerament^®^ visualized by means of CTVox software: (A) total volume, (B) cross-section and (C) only the radiopaque phase. Colors legend: CSD matrix in purple, and radiopaque element in green. Figure S6. Pore size distributions of the investigated cements calculated through individual 3D analyses. Figure S7. Qualitative representations of the pore distribution throughout the cement volumes obtained by using the Micro-CT software. Figure S8. Calcium ion release kinetics from the optimized composite cement formulation with (orange line) and without (blue line) ICOS_PLGA.
