# Supplementary material for: Optimization of an Injectable, Resorbable, Bioactive Cement Able to Release the Anti-Osteoclastogenic Biomolecule ICOS-Fc for the Treatment of Osteoporotic Vertebral Compression Fractures
Source: Biomolecules. 2023 Jan 2;13(1):94. doi: 10.3390/biom13010094 (PMC9855932; doi:10.3390/biom13010094)
Supplement: Supplementary file 1 [file biomolecules-13-00094-s001.zip › biomolecules-2036644-supplementary.pdf]

biomolecules

MDPI

Supporting Information

Optimization of an Injectable, Resorbable, Bioactive Cement Able to Release the Anti-Osteoclastogenic Biomolecule ICOS-Fc for the Treatment of Osteoporotic Vertebral Compression Fractures

Federica Banche-Niclot [1], Ilaria Corvaglia [1], Caterina Cavalera [1], Elena Boggio [2,3], Casimiro Luca Gigliotti [2,3], Umberto Dianzani [3], Antzela Tzagiollari [4,5], Nicholas Dunne [4,5], Antonio Manca [6], Sonia Fiorilli [1,7] and Chiara Vitale-Brovarone [1,7,*]

[1] Department of Applied Science and Technology, Politecnico di Torino, Corso Duca Degli Abruzzi 24, 10129 Torino, Italy
[2] NOVAICOS s.r.l.s., Via Amico Canobio 4/6, 28100 Novara, Italy
[3] Department of Health Sciences, Università del Piemonte Orientale, Via Solaroli 17, 28100 Novara, Italy
[4] Centre for Medical Engineering Research, School of Mechanical and Manufacturing Engineering, Dublin City University, D09 NA55 Dublin, Ireland
[5] Biodesign Europe, Dublin City University, D09 NA55 Dublin, Ireland
[6] Department of Radiology, Candiolo Cancer Institute, FPO-IRCCS, 10060 Torino, Italy
[7] National Interuniversity Consortium of Materials Science and Technology, RU Politecnico di Torino, 50121 Firenze, Italy
* Correspondence: chiara.vitalebrovarone@polito.it.

	Density	Size	Morphology
ZrO_2 (commercial available)	5.7 cm^3/g	100-250 nm	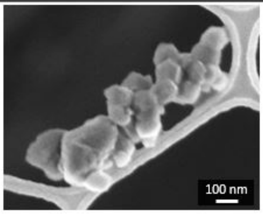

	Specific Surface Area (SSA)	Pore Volume	Density	Size	Morphology
Sr-MBG-SG	650 m^2/g	0.7 cm^3/g	0.8 cm^3/g	100-500 nm	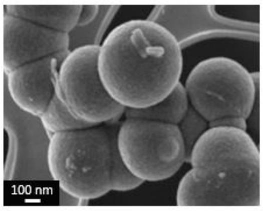
Sr-MBG -SD	100 m^2/g	0.1 cm^3/g	2.2 cm^3/g	1-5 µm	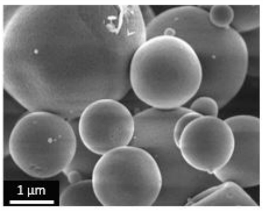

Figure S1. Characteristics and morphology of the commercially available ZrO2 nanoparticles exploited to impart radiopacity to the cement as well as of the two types of Sr-MBGs separately included within the formulation to impart bioactive and pro-osteogenic features.

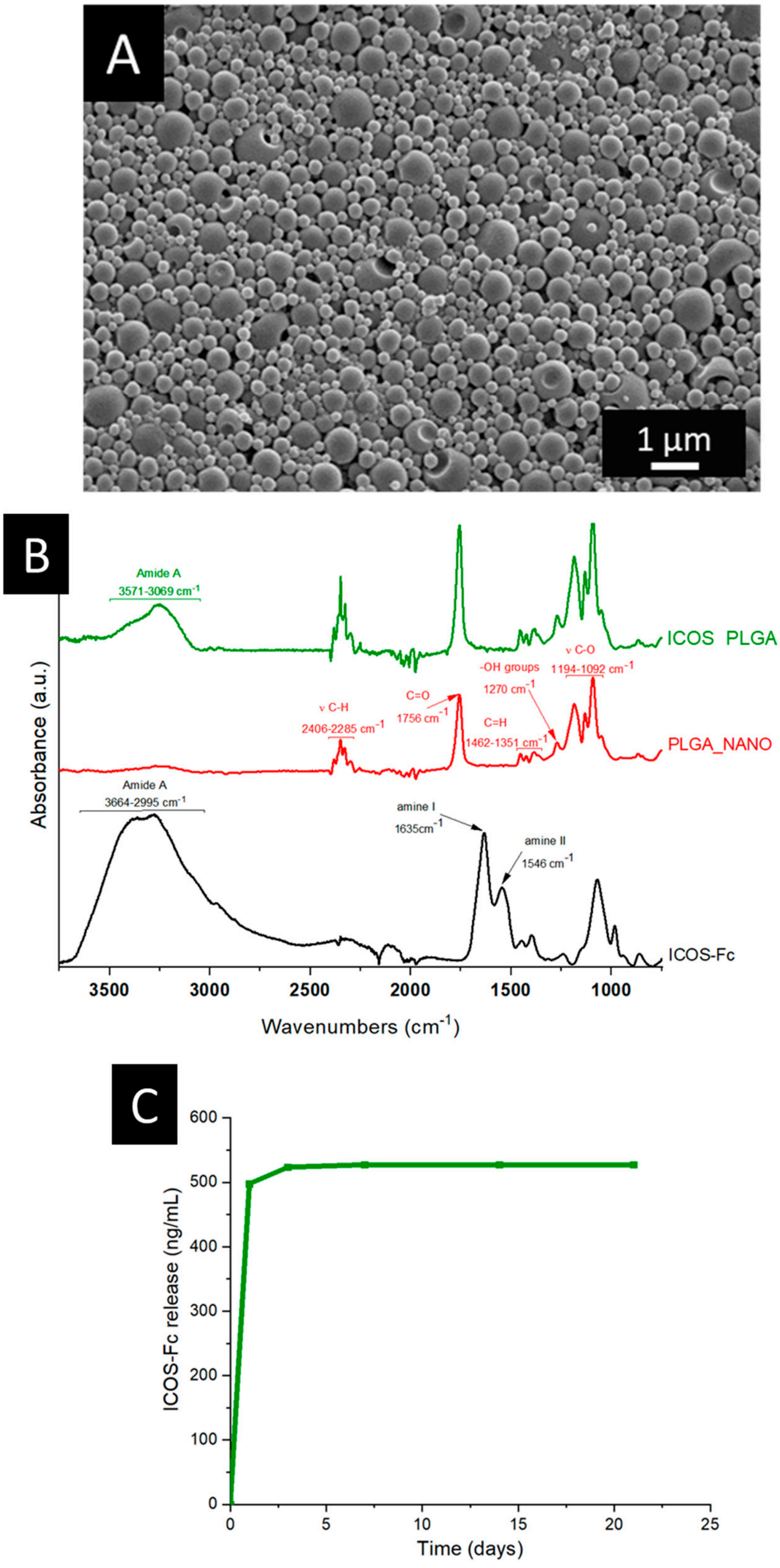

Figure S2. ICOS_PLGA nanoparticles characterization: (A) FE-SEM images of ICOS_PLGA nanoparticles (25,000×). (B) ATR-FTIR spectra of ICOS-Fc (black line), unloaded PLGA nanoparticles (red line) and ICOS_PLGA carriers (green line). (C) Cumulative amount of released ICOS-Fc from PLGA nanoparticles as a function of time determined by means of Micro BCA Protein Assay.

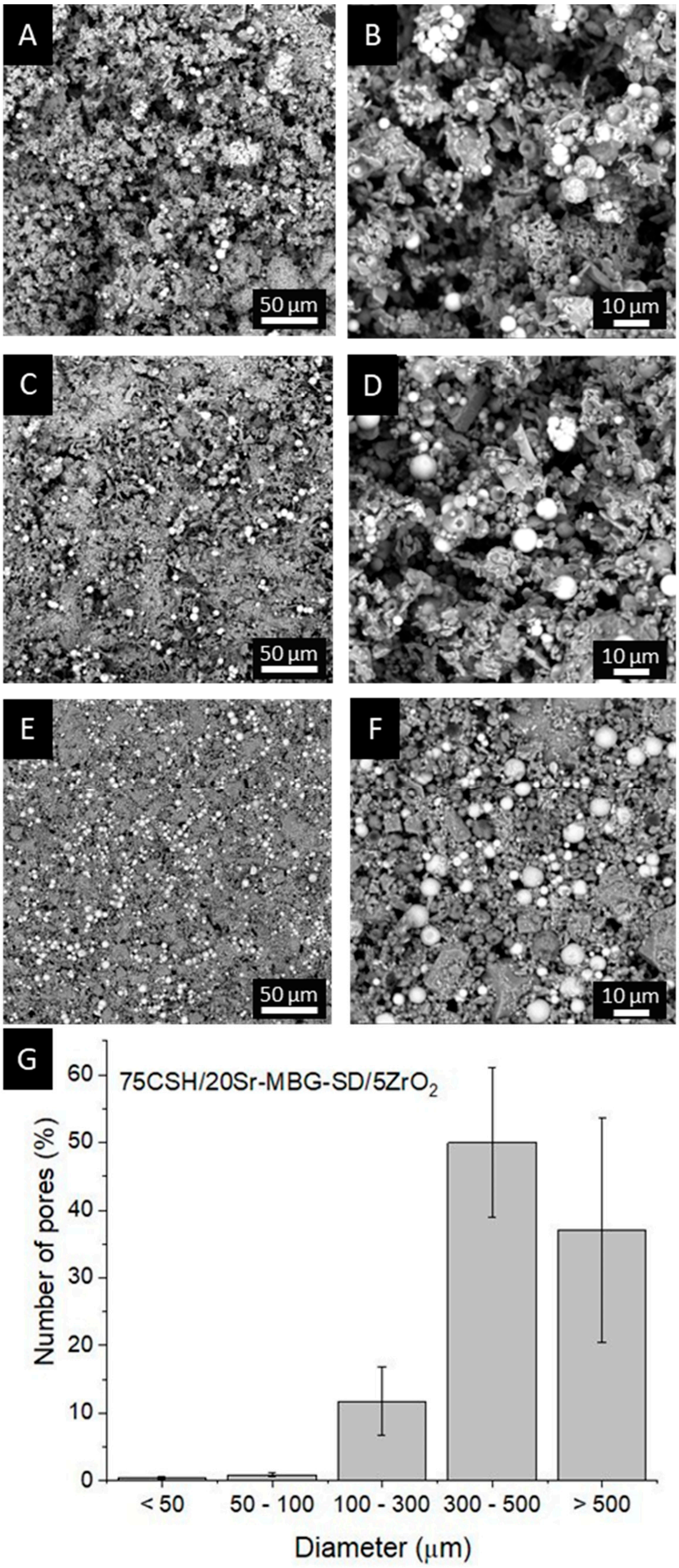

Figure S3. SEM images of cement formulations containing Sr-MBG-SD acquired at 1,000× and 3,000×: (A-B) 75CSH/20Sr-MBG-SD/5ZrO_2, (C-D) 80CSH/15Sr-MBG-SD/5ZrO_2, (E-F) 85CSH/10Sr-MBG-SD/5ZrO_2. (G) Pore size distribution of the 75CSH/20Sr-MBG-SD/5ZrO_2 formulation.

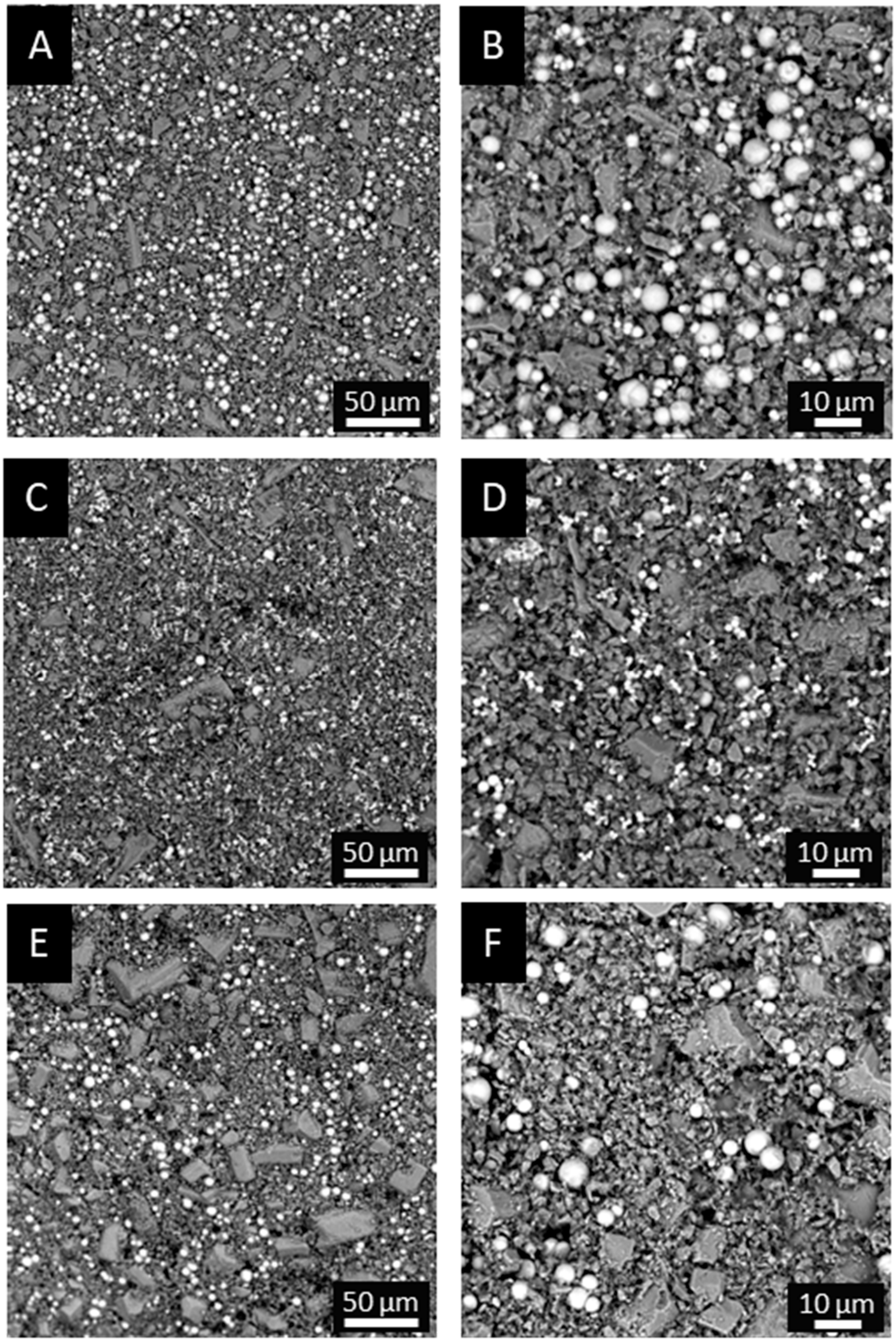

Figure S4. SEM images of cement formulations containing Sr-MBG-SG acquired at 1,000× and 3,000×: (A-B) 75CSH/20Sr-MBG-SG/5ZrO_2, (C-D) 80CSH/15Sr-MBG-SG/5ZrO_2 and (E-F) 85CSH/10Sr-MBG-SG/5ZrO_2.

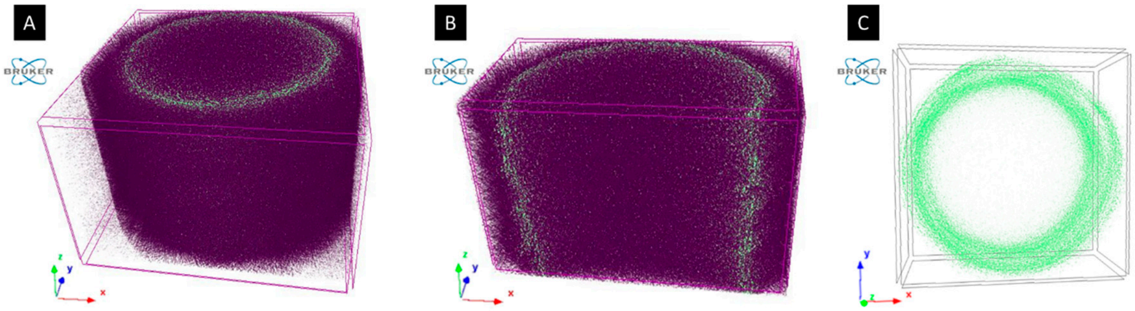

Figure S5. Radiopacity distribution of Cerament® visualized by means of CTVox software: (A) total volume, (B) cross-section and (C) only the radiopaque phase. Colors legend: CSD matrix in purple, and radiopaque element in green.

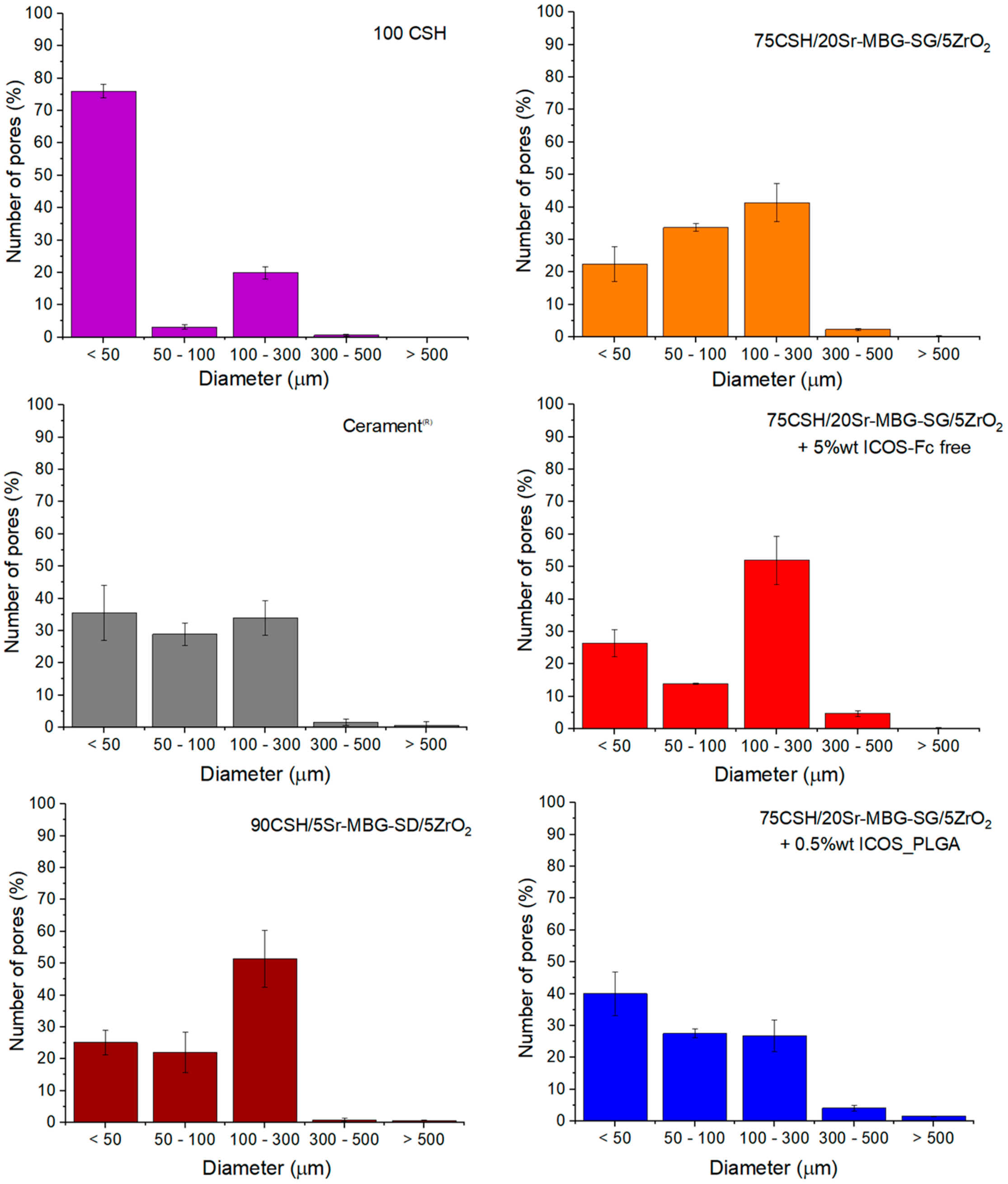

Figure S6. Pore size distributions of the investigated cements calculated through individual 3D analyses.

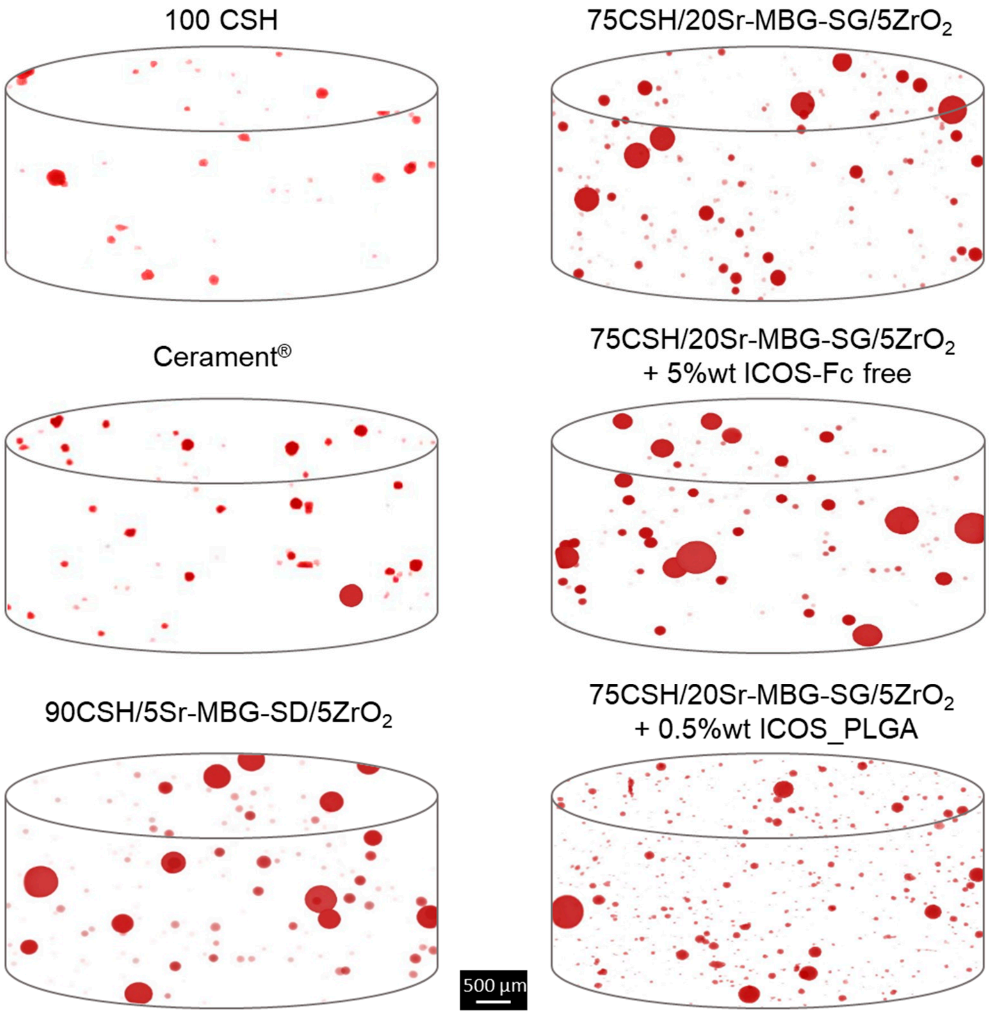

Figure S7. Qualitative representations of the pore distribution throughout the cement volumes obtained by using the Micro-CT software.

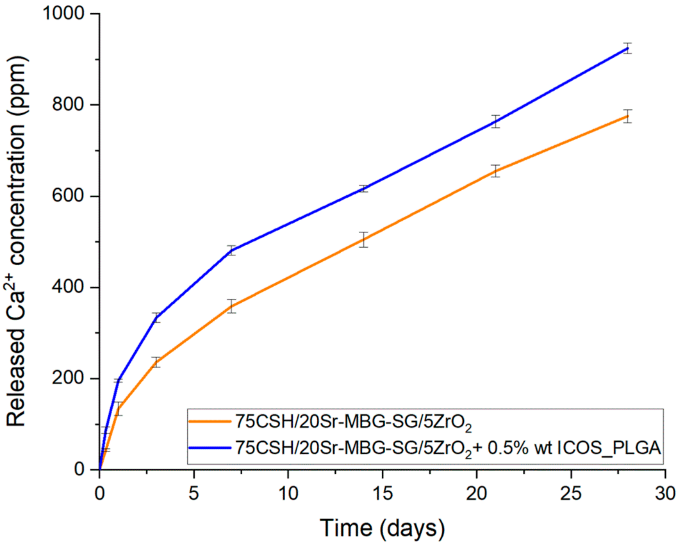

Figure S8. Calcium ion release kinetics from the optimized composite cement formulation with (orange line) and without (blue line) ICOS_PLGA.
